# Innate Immune Tolerance Regulates Microglia Response to Aβ Oligomers

**DOI:** 10.1111/jnc.70341

**Published:** 2026-01-02

**Authors:** Rafaela Rodrigues Valerio, Áquila Rodrigues Santos, Ana Helena Larangeira Nóbrega, Raquel Martins, Fernanda G. De Felice, Sergio T. Ferreira, Wilson Savino, Adriana Bonomo, Andressa Bernardi, Rudimar Luiz Frozza

**Affiliations:** ^1^ Laboratory on Thymus Research, Oswaldo Cruz Institute Oswaldo Cruz Foundation, FIOCRUZ Rio de Janeiro Brazil; ^2^ Laboratory of Inflammation, Oswaldo Cruz Institute Oswaldo Cruz Foundation, FIOCRUZ Rio de Janeiro Brazil; ^3^ Institute of Medical Biochemistry Leopoldo de Meis Federal University of Rio de Janeiro Rio de Janeiro Brazil; ^4^ Centre for Neuroscience Studies, Department of Biomedical and Molecular Sciences and Department of Psychiatry Queen's University Kingston Ontario Canada; ^5^ D'Or Institute for Research and Education Rio de Janeiro RJ Brazil; ^6^ National Institute of Science and Technology for Translational Neuroscience Rio de Janeiro RJ Brazil; ^7^ Institute of Biophysics Carlos Chagas Filho Federal University of Rio de Janeiro Rio de Janeiro RJ Brazil; ^8^ National Institute of Science and Technology on Neuroimmunomodulation Rio de Janeiro RJ Brazil; ^9^ Rio de Janeiro Research Network on Neuroinflammation/FAPERJ Rio de Janeiro RJ Brazil; ^10^ INOVA‐IOC Network on Neuroimmunomodulation Rio de Janeiro RJ Brazil; ^11^ Rio Network of Innovation in Nanosystems for Health ‐ Nanohealth/FAPERJ Rio de Janeiro RJ Brazil

**Keywords:** Alzheimer's disease, cytokines, immune tolerance, innate immune memory, microglia, neuroinflammation

## Abstract

Microglia are the main innate immune cells residing in the brain parenchyma. Their activation and resulting neuroinflammation have emerged as major pathogenic mechanisms in neurodegenerative disorders, particularly in Alzheimer's disease (AD). The accumulation of amyloid‐β oligomers (AβOs) and microglia activation play crucial roles in the pathogenesis of AD. In a second vein, the development of innate immune memory in response to different stimuli is a vital mechanism that enables microglia to adjust their response to subsequent inflammatory challenges. While there is increasing evidence that repeated bouts of peripheral inflammation lead to training or tolerance in microglia, the impact of tolerance on the inflammatory response induced by AβOs remains to be determined. In this study, we investigated whether lipopolysaccharide (LPS)‐induced tolerance affects microglial responses to AβOs. For that, organotypic hippocampal cultures were repeatedly challenged with LPS before being exposed to AβOs. We measured cytokine levels and evaluated changes in microglial activation and morphology following exposure of cultures to AβOs. A significant decrease in cytokine production was observed when hippocampal slice cultures were repeatedly challenged with LPS. Interestingly, microglial activation and the resulting inflammatory response induced by AβOs were prevented when these cultures had been previously challenged with LPS. Moreover, the changes in microglial morphology and cytokine production resulting from repeated LPS stimulation were associated with reduced activation of nuclear factor kappa B (NF‐κB). These results indicate that preconditioning microglia with LPS induces a physiological immune tolerance response rather than pathological inflammation, which may have implications for developing therapeutic strategies for AD aimed at modulating innate immune memory.

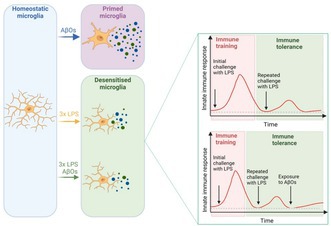

## Graphical abstract

Microglia are the primary innate immune cells residing in the brain parenchyma, and their activation in Alzheimer's disease (AD) results in increased neuroinflammation. The development of innate immune memory in response to various stimuli is a crucial mechanism that allows microglia to tailor their responses to future inflammatory challenges. Our study shows that preconditioning microglia with lipopolysaccharide (LPS) triggers a physiological immune tolerance response rather than causing pathological inflammation, thereby reducing the increased cytokine production caused by amyloid‐β oligomers (AβOs). This finding may have important implications for developing therapeutic strategies for AD that focus on modulating innate immune memory.

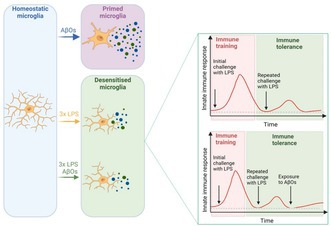

**Abbreviations**
AD: Alzheimer's disease
ANOVA: analysis of variance
AβOs: amyloid‐β peptide oligomers
BCA: bicinchoninic acid
BSA: bovine serum albumin
CBP: CREB‐binding protein
CNS: central nervous system
COX‐2: cyclooxygenase‐2
CREB: cAMP response element‐binding protein
DMSO: anhydrous dimethyl sulfoxide
GFAP: glial fibrillary acidic protein
HBSS: hanks' balanced salt solution
HDAC2: histone deacetylase 2
HFIP: 1,1,1,3,3‐hexafluoro‐2‐propanol
HPLC: high‐performance liquid chromatography
ICER: inducible cAMP early repressor
IFN‐β: interferon beta
IL: interleukin
IL‐1R1: type 1 interleukin‐1 receptor
IL‐1β: interleukin‐1 beta
IκBα: inhibitor of nuclear factor‐kappa B
JNK: Jun N‐terminal kinase
LDH: lactate dehydrogenase
LPS: lipopolysaccharide
Mino: minocycline
MSK 1/2: mitogen‐and stress‐activated protein kinase
p65 NF‐κB: p65 subunit of nuclear factor‐kappa B
PAMPs: pathogen‐associated molecular patterns
PBS: phosphate‐buffered saline
PSD‐95: postsynaptic density protein 95
RIPA: radio‐immunoprecipitation assay buffer
RRID: Research Resource Identifier
TBS: tris buffered saline
TLR4: toll‐like receptor 4
TREM2: triggering receptor expressed on myeloid cells 2

## 1 Introduction

Alzheimer's disease (AD) is a progressive neurodegenerative disorder characterized by memory loss (Cummings 2002; Soria Lopez et al. 2019). Currently affecting more than 1.7% of the global population, AD is the most common cause of dementia (Tahami Monfared et al. 2022). Because aging is the main risk factor, its prevalence keeps growing along with life expectancy, making it a major global public health concern.

Synaptic deterioration as a consequence of brain buildup of amyloid plaques and tangles, and the accumulation of soluble oligomers of amyloid‐β peptide (AβOs) plays a pivotal role in the pathogenesis of AD (Ferreira et al. 2015; Palop and Mucke 2010; Abubakar et al. 2022; Rummel and Butterfield 2022). Furthermore, it is currently accepted that neuroinflammation is a core hallmark of the neurodegenerative process in AD (Castro‐Gomez et al. 2019). These features are accompanied by exacerbated glial activation and neuronal loss.

Microglia, the resident macrophages of the central nervous system (CNS), are key elements in the cerebral immune milieu, and their aberrant activation may trigger a cascade of inflammatory responses. Additionally, over the last two decades, several genome‐wide association studies and whole genome sequencing analyses have found > 51% enrichment in AD‐risk genes related to innate immune system pathways (Sims et al. 2017; Lambert et al. 2009; Guerreiro et al. 2013). These findings are associated with compelling evidence implicating brain microglia as important players in the development and progression of AD (Efthymiou and Goate 2017; Fan et al. 2017).

Microglia recognize different forms of misfolded proteins and initiate subsequent immune responses, mediating neuroinflammation and neuron–glia crosstalk. Despite their central role in maintaining brain homeostasis and synaptic integrity, aberrant, and prolonged microglial activation has harmful effects on the CNS (Chagas et al. 2020). Previous studies indicate that AβOs induce microglial activation and production of pro‐inflammatory cytokines (including TNF‐α, IL‐1β, IL‐6, and IL‐8), chemokines, and reactive oxygen and nitrogen species, all of which could lead to neuronal dysfunction and death (Lourenco et al. 2013; Ledo et al. 2013, 2016; Muzio et al. 2021). In line with these findings, clinical studies have shown that inflammatory markers are increased in both the plasma and brain of AD patients (Greenhalgh et al. 2020; Leng and Edison 2021; Swardfager et al. 2010; Brosseron et al. 2014).

Microglia exposure to inflammatory stimuli induces their activation and broad, long‐lasting metabolic/epigenetic reprogramming (Ferreira et al. 2022; Zhang et al. 2022). These changes allow that, when microglia encounter a subsequent inflammatory stimulus, they show altered responsiveness, characterizing a phenomenon of innate immune memory (Wendeln et al. 2018; Divangahi et al. 2021; Neher and Cunningham 2019; Haley et al. 2019). Such innate immune memory includes immune training and immune tolerance, which refer to enhanced or suppressed immune responses by myeloid cells upon secondary stimulation, respectively (Divangahi et al. 2021), and can be elicited by heterologous *stimuli* (Arts et al. 2018; Saeed et al. 2014).

A growing body of evidence demonstrates that systemic inflammation modifies microglial activation, causes synapse dysfunction and loss, and increases brain Aβ loads (Lee et al. 2008; Go et al. 2016; Wendeln et al. 2018; Tejera et al. 2019; Knopp et al. 2022). Interestingly, several studies suggest that modulating the activation of immune cells may protect against neuroinflammatory disorders, such as AD (Wendeln et al. 2018; Yang et al. 2023). Repeated exposure of innate immune cells to a sublethal dose of endotoxins, such as lipopolysaccharide (LPS), can induce endotoxin tolerance (Seeley and Ghosh 2017), a type of innate immune tolerance for memory. Cells that have attained innate immune tolerance are resistant to subsequent inflammatory challenges, as evidenced by decreased production of inflammatory factors (Wendeln et al. 2018; Twayana et al. 2019; Divangahi et al. 2021; Charoensaensuk et al. 2024). Previous studies have shown that preconditioning with low‐dose LPS induces suppression of proinflammatory mediators (Antonietta Ajmone‐Cat et al. 2013) and improves cognitive decline in animals (Wendeln et al. 2018; Lajqi et al. 2019; Mizobuchi et al. 2020; Charoensaensuk et al. 2024). However, to the best of our knowledge, studies aimed to investigate whether eliciting innate immune tolerance attenuates microglial responses to AβOs have not been reported.

Herein, we show that the induction of immune tolerance in microglia attenuates inflammation and microglial activation induced by AβOs. We demonstrate that organotypic hippocampal slice cultures exposed repeatedly to LPS show progressive decreases in the release of TNF‐α, IL‐1β, IL‐6, and IL‐10. Remarkably, previous exposure of cultures to LPS reduces microglial activation, evidenced by decreased expression of activation markers and changes in morphology, also abrogating the activation of the nuclear factor kappa B (NF‐κB) pathway and production of inflammatory cytokines induced by AβOs. Collectively, our findings suggest that chronic, low‐intensity stimulation with LPS leads to immune tolerance in microglia, blocking the neuroinflammation caused by AβOs.

## 2 Methods

### 2.1 Preparation of Aβ Oligomers (AβOs)

Oligomers were prepared from a synthetic Aβ1‐42 peptide (cat. no. 155178‐13‐5, Echelon Biosciences, Salt Lake, USA) as originally described (Lambert et al. 1998; Figueiredo et al. 2013) and were routinely characterized by size exclusion chromatography and occasionally by western blot (Figueiredo et al. 2013; Lourenco et al. 2019). Briefly, the peptide was first solubilized in 1,1,1,3,3‐hexafluoro‐2‐propanol (HFIP, cat. no. 920‐66‐1, Merck, Darmstadt, Germany), which was then evaporated to form dried films. These films were subsequently dissolved in sterile, anhydrous dimethyl sulfoxide (DMSO, cat. no. 67‐68‐5, Sigma‐Aldrich, St. Louis, MO, USA) to obtain a 5 mM solution. This solution was diluted to 100 μM in ice‐cold sterile phosphate‐buffered saline (PBS, cat. no. 7558‐80‐7, Sigma‐Aldrich, St. Louis, MO, USA) and incubated for 16 h at 4°C. Thereafter, the preparation was centrifuged at 14000*g* for 10 min at 4°C to remove insoluble aggregates (protofibrils and fibrils), and the supernatants containing soluble AβOs were stored at 4°C and used within 48 h of preparation. The protein concentration was determined using the bicinchoninic acid assay (BCA, cat. no. A55860, Pierce—Thermo Scientific). The preparations were routinely characterized by high‐performance liquid chromatography (HPLC) size exclusion chromatography under non‐denaturing conditions (De Felice et al. 2007; Lourenco et al. 2019).

### 2.2 Animals

Male *Wistar* rats (RRID: MGI: 5657554) (7–9 days old) were obtained from the animal facility at the Instituto de Ciências e Tecnologia em Biomodelos (ICTB/Fiocruz). All experimental procedures were performed according to the recommendations of the Guide for the Care and Use of Laboratory Animals of the National Council for Animal Experimentation (Brazil) and were approved by the Animal Use Ethics Committee of Oswaldo Cruz Institute/Fiocruz (license L‐018/2020‐A1). A total of 111 male pups at postnatal Day 7–9 were used in this study. The number of animals required for each experiment was estimated based on the previous experience of the authors with the impact of Aβ oligomers and fibrils on neurotoxicity, neuroinflammation, and synaptic proteins (Simões‐Pires et al. 2021; Hoppe et al. 2013; Frozza et al. 2009). The litter was housed with the dam in microisolator cages (type IV) with a maximum of 10 animals per cage. They had free access to food and water and were kept on a 12‐h light–dark cycle (lights on from 07:00 am to 07:00 pm). The room temperature was maintained at 20°C (±1°C) with a humidity level of 50%–60%. The average body weight of pups used in this study was 15 g.

### 2.3 Organotypic Hippocampal Slice Culture

Organotypic hippocampal tissue cultures were prepared according to the method described by Stoppini and coworkers (Stoppini et al. 1991), with some modifications (Frozza et al. 2009). Briefly, postnatal Day 7–9 male Wistar rats were anesthetized with isoflurane (Cristália; São Paulo, Brazil) at 2.5% in 100% oxygen (1.0 L/min) and decapitated; their brains were quickly removed from the skull and washed with ice‐cold Hanks' balanced salt solution (HBSS, cat. no. H4641, Sigma‐Aldrich, St. Louis, MO, USA). Both hippocampi were dissected and cut into 400 μm‐thick slices using a McIlwain tissue chopper (cat. no. 10180, Ted Pella Inc., Redding, CA, USA). Slices were separately immersed in ice‐cold HBSS and arranged onto organotypic inserts of standard six‐well cell culture plates (cat. no. PICM03050, Millicell, Millipore). In every experiment, each organotypic insert subjected to a specific experimental treatment contained two hippocampal slices from each of three distinct animals (six slices per insert), and the result from each insert generated a single data point within a single experiment. The number of independent experiments (N) is indicated in the legend of each figure.

The slices were maintained in culture in an incubator at 37°C with 5% CO_2_ using an interface method with 1 mL of medium supplying the undersurface of the insert. The culture medium consisted of 50% minimum essential medium (cat. no. 12492013, Invitrogen, Grand Island, NY, USA), 25% HBSS (cat. no. 14025092, Invitrogen), and 25% heat‐inactivated horse serum (cat. no. 16050114, Invitrogen), supplemented with 36 mM D‐glucose (cat. no. 50‐99‐7, Sigma‐Aldrich), 25 mM HEPES (cat. no. 7365‐45‐9, Sigma‐Aldrich), 4 mM NaHCO_3_ (cat. no. 144‐55‐8, Merck), and 1% penicillin/streptomycin/amphotericin (cat. no. 15240062, Invitrogen), pH 7.3. The first medium change was performed 30 min after slice preparation, and this process was repeated every 3–4 days. The slices were maintained for 14 days in vitro before experiments.

### 2.4 Tolerance Induction and AβOs Exposure

To induce innate immune memory translating into a functional state in which microglia suppress their response to a subsequent inflammatory challenge, organotypic hippocampal slice cultures were incubated with or without LPS from *Escherichia coli* (0127: B8, cat. no. L3129, Specification: PRE.2.ZQ5.10000001349, Sigma‐Aldrich) at 5 μg/mL (Antonietta Ajmone‐Cat et al. 2013) starting from the 11th day in culture added to culture medium every 24 h for 3 days. Unstimulated cultures had the medium changed every 24 h for 3 days. The collected medium every 24 h was kept at −80°C for subsequent analysis of inflammatory cytokines and cell viability. Twenty‐four hours after the last LPS challenge, the cultures were exposed to 500 nM of AβOs, added to the new culture medium, for 24 h. After 24 h of exposure of the cultures to AβOs, the medium was collected to assess levels of inflammatory cytokines and cell viability, while the slices were used for further biochemical and morphological analyses.

### 2.5 Microglia Inhibition or Depletion

To evaluate whether the innate immune response was mediated by microglia, we added 10 μM of minocycline (cat. no. AM11587, Biosynth, USA) continuously to the medium of specific wells, starting on the seventh day of culture and continuing until the end of the experiment to inhibit microglial activation. Additionally, some organotypic hippocampal slice cultures were treated with 10 μM of PLX5622 (cat. no. 1303420‐67‐8, Cayman Chemical, USA), a CSF1 receptor inhibitor that can efficiently deplete microglia. PLX5622 was also continuously added to the medium of specific wells from the seventh day of culture until the conclusion of the experiment. To confirm the successful depletion of microglia, we used immunofluorescence to quantify the number of microglia in the slices 24 h after exposure to AβOs.

### 2.6 Viability Assessment of Organotypic Hippocampal Slice Cultures

Organotypic hippocampal slice culture viability along the time in culture and after LPS challenges and AβOs exposure was assessed by using the CyQUANT LDH Cytotoxicity Assay (Thermo‐Scientific, cat. no. C20300), which provides a quantification of cytotoxicity/cytolysis based on the measurement of lactate dehydrogenase (LDH) activity released from damaged cells into the culture medium. As an internal control for cell death (maximum LDH activity control), slices were incubated with lysis buffer (supplied for CyQUANT LDH Cytotoxicity Assay) for 45 min, and then the LDH activity was determined in the culture medium. Changes in the relative amount of LDH in the culture medium were expressed as a percentage of the maximum LDH activity control, which was considered 100% of cell death.

### 2.7 Immunofluorescence and Confocal Microscopy

To examine microglial morphology and microglial inhibition or depletion efficacy, organotypic hippocampal slice cultures were processed according to the methodology described by Gogolla and coworkers (Gogolla et al. 2006). Briefly, 24 h after the exposure to AβOs, the slices were fixed with 4% paraformaldehyde in 0.01 M PBS for 3 h at room temperature, post‐fixed for 5 min in cooled 20% methanol in PBS, and incubated overnight in permeabilization solution (0.5% Triton X‐100 in PBS). The slices were incubated at 4°C for 24 h in blocking solution (20% BSA in PBS), washed three times with PBS, detached from the insert membrane, transferred to a 24‐well plate, and then incubated overnight at 4°C with an anti‐Iba‐1 antibody (RRID: AB_3094956) (1: 400, FUJIFILM Wako Chemicals, Richmond, VA, USA) diluted in 5% BSA in PBS followed by an Alexa‐Fluor 647‐conjugated donkey anti‐rabbit IgG (RRID: AB_2866496) (1: 500, Invitrogen) for 3–4 h at room temperature. Afterward, the slices were washed three times with PBS and incubated for 5 min with Hoescht 33342 (1: 1000 in PBS, Invitrogen). The sections were mounted on gelatin‐coated slides, cover slipped with Prolong Gold Antifade (Invitrogen), and sealed with nail polish. Immunolabeled microglia were imaged with a confocal microscope (Leica TCS SP8 laser scanner microscopy, Wetzlar, Germany) with 20× (NA 0.75; Leica) and 63× (NA 1.40; Leica) oil‐submersion magnifications. Each image obtained was a z‐stack of 20–25 (4 μm depth) sections, and three independent fields from each slice were selected for analysis. Images were thresholded based on control slices immunostained with secondary antibodies alone, run in parallel with each experiment. The fluorescence intensity, number of Iba‐1‐positive cells, circularity, area and length of the soma, aspect ratio, and the number of branches of Iba‐1‐positive cells were determined with NIH ImageJ software. Image analyses were conducted by an experimenter, blinded to the different groups.

### 2.8 Cytokine Determination

The culture medium was collected every 24 h after the challenge with LPS, as well as after 24 h of exposure to AβOs, and stored immediately at −80°C. The levels of the cytokines TNF‐α (cat. no. DY510‐05), IL‐1β (cat. no. DY501‐05), IL‐6 (cat. no. DY506‐05), and IL‐10 (cat. no. DY522‐05) were measured according to the manufacturer's instructions (DuoSet; R&D Systems, Minneapolis, MN, USA). Values were expressed as picograms per milliliter.

### 2.9 Western Blotting

Twenty‐four hours after the incubation period with the AβOs, the slices were lysed using protein extraction buffer (RIPA, cat. no. P0013B, Thermo‐Fisher Scientific) containing phosphatase and protease inhibitors (cat. no. 78446 Pierce–Thermo‐Fisher Scientific). Protein concentration was determined using the bicinchoninic acid (BCA) assay kit. Denatured protein was diluted to an equal concentration, prepared with 4× Laemmli Sample Buffer (cat. no. 1610747, Bio‐Rad), and 30 μg of protein was resolved on 14% polyacrylamide gels with Tris/glycine/SDS buffer run at 220 V for approximately 90 min at room temperature. The gel was electroblotted onto Hybond ECL nitrocellulose (cat. no. GE10600002—Amersham) using 25 mM Tris, 192 mM glycine, and 20% (v/v) methanol (pH 8.3) at 350 mA for 90 min at 4°C. The membranes were blocked with 5% BSA in TBS‐T solution for at least 1 h at room temperature. The primary antibodies used were against Iba‐1 (RRID: AB_3094956, 1: 1000, FUJIFILM Wako Chemicals, Richmond, VA, USA), TREM‐2 (RRID: AB_3101799, 1: 1000, Merck Millipore), GFAP (RRID: AB_2799321, 1: 1000, Cell Signaling Technology), JNK (RRID: AB_2141027, 1: 1000, Cell Signaling Technology), p‐JNK (RRID: AB_823588, 1: 1000, Cell Signaling Technology), COX‐2 (RRID: AB_2084968, 1: 1000, Cell Signaling Technology), Synaptophysin (RRID: AB_1904154, 1:1000, Cell Signaling Technology), PSD95 (RRID: AB_561221, 1: 1000, Cell Signaling Technology), GFAP (RRID: AB_2109642, 1:1000, Sigma‐Aldrich), NF‐κB (RRID: AB_3086883, 1:1000, Cell Signaling Technology), p‐NF‐κB (RRID: AB_2341216, 1: 1000, Cell Signaling Technology), RelB (RRID: AB_2797727, 1: 1000, Cell Signaling Technology), IκB (RRID: AB_2800211, 1: 1000, Cell Signaling Technology), p‐IκB (RRID: AB_10758977, 1:1000, Cell Signaling Technology), Acetyl‐Histone H3 (Lys9) (RRID: AB_310308, 1:1000, Merck Millipore), Acetyl‐Histone H4 (Lys16) (RRID:AB_310525, 1:1000, Merck Millipore), Histone H3 (RRID: AB_2756816, 1: 1000, Cell Signaling Technology), Histone H4 (RRID: AB_1147658, 1: 1000, Cell Signaling Technology), HDAC2 (RRID: AB_2756828, 1: 1000, Cell Signaling Technology), β‐actin (RRID: AB_2223172, 1:1000, Cell Signaling Technology), and β‐tubulin (RRID: AB_2827403, 1: 1000, Sigma‐Aldrich). The antibodies were diluted in a blocking solution and incubated with the membranes overnight at 4°C. After incubation with peroxidase‐conjugated secondary anti‐mouse or anti‐rabbit IgG (1:5.000 in blocking solution; Invitrogen) for 1 h at room temperature, the membranes were washed with T‐TBS and the bands were digitally visualized using chemiluminescent substrate (SuperSignal West Femto Maximum Sensitivity substrate, cat. no. 34096—Pierce‐Thermo Fisher Scientific) in a ChemiDoc imager. Images were captured using Quantity One software (Bio‐Rad), and optical density determination was performed on ImageJ software. The results represent the ratio of the phosphorylated protein to total protein or total protein to β‐tubulin or β‐actin protein.

### 2.10 Statistical Analysis

GraphPad Prism 9.0 (GraphPad, La Jolla, CA) was used for statistical analysis. All data are represented as the mean and standard error of the mean (mean ± SEM). The data were tested with Grubb's outlier test (*α* = 0.05), and no data points were excluded. To check for normal distribution, the Shapiro–Wilk test was used. Two‐way analysis of variance (ANOVA) followed by the Holm‐Sidák test was used every time multiple comparisons were performed. *p*‐values were considered as follows: *p* > 0.05 = ns; *p ≤ 0.05; **p ≤ 0.01; ***p ≤ 0.001; ****p ≤ 0.0001. The significance levels and the number of animals or independent experiments (N) are indicated in the legend of each figure.

## 3 Results

### 3.1 Chronic Stimulation of Organotypic Hippocampal Slice Cultures With LPS Induces Tolerance in Microglia

**FIGURE 1:**
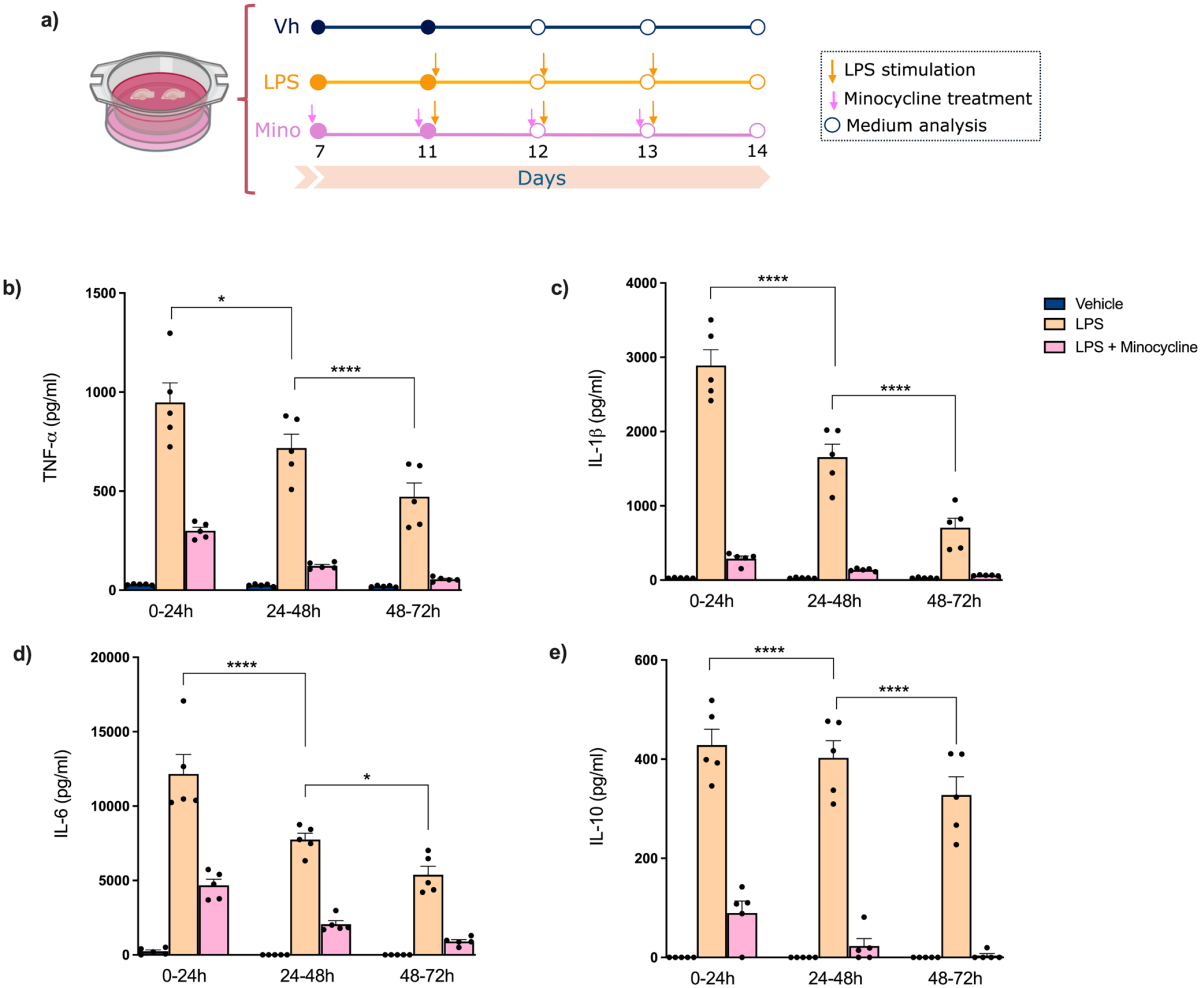
Repeated LPS challenge results in decreased levels of inflammatory cytokines in organotypic hippocampal slice cultures. Slices were cultured in the presence of 5 μg/mL LPS added after collecting and replacing the medium every 24 h (at time points *t*_0_, *t*_24_, *t*_48_) from Day 11 to 14. To inhibit microglia, minocycline (10 μM) was continuously added to the culture medium starting from the seventh day until the end of the culture period. After the first, second, and third LPS stimulations, the culture medium was collected and analyzed for cytokine levels. (a) Schematic timeline illustrating the repeated LPS stimulation of the organotypic hippocampal slice cultures. (b) TNF‐α, (c) IL‐1β, (d) IL‐6, and (e) IL‐10 levels in the culture medium after each LPS challenge (*n* = 5 independent cultures per group; 3 animals/culture). The results are presented as the mean ± standard error of mean (mean ± SEM). The data were analyzed using a two‐way analysis of variance (ANOVA) followed by the Holm‐Sidák test. *p < 0.05; ****p < 0.0001.

To investigate whether a chronic low‐intensity inflammatory stimulus could lead to the development of innate immune memory, LPS (5 μg/mL) was added to the fresh medium of organotypic hippocampal cultures every 24 h over a 72 h period (Figure 1a). To assess whether the inflammatory response was mediated by microglia, minocycline (Mino) was added to the medium starting 96 h before the first LPS challenge and continued until the end of the LPS exposure to inhibit microglial activation (Han et al. 2019) (Figure 1a). We confirmed that exposure of cultures to LPS resulted in increased levels of TNF‐α [*F* (8, 36) = 53.63; *p* < 0.0001], IL‐1β [*F* (8, 36) = 95.97; *p* < 0.0001], IL‐6 [*F* (8, 36) = 64,87; *p* < 0.0001], and IL‐10 [*F* (8, 36) = 71,93; *p* < 0.0001] (Figure 1b–e) within the first 24 h. Interestingly, the cytokine production in response to subsequent LPS challenges decreased after the initial challenge (Figure 1b–e). Noteworthy, the attenuation of microglia activation by Mino prevented the increases in proinflammatory cytokines (Figure 1b–e). Similarly, when microglia were depleted using PLX5622, the production of cytokines was inhibited, including TNF‐α [*F* (8, 9) = 3947; *p* = 0.0281], IL‐1β [*F* (8, 9) = 16,97; *p* < 0.0001] and IL‐6 [*F* (8, 9) = 2077; *p* = 0.1485] (Figure S2). These findings indicate that repeated stimulation with LPS leads to a reduction in the production of inflammatory cytokines by microglial cells, suggesting the development of immune tolerance.

### 3.2 Tolerant Microglia Exhibit Reduced Production of Proinflammatory Cytokines After Exposure to AβOs in Organotypic Hippocampal Slice Cultures

**FIGURE 2:**
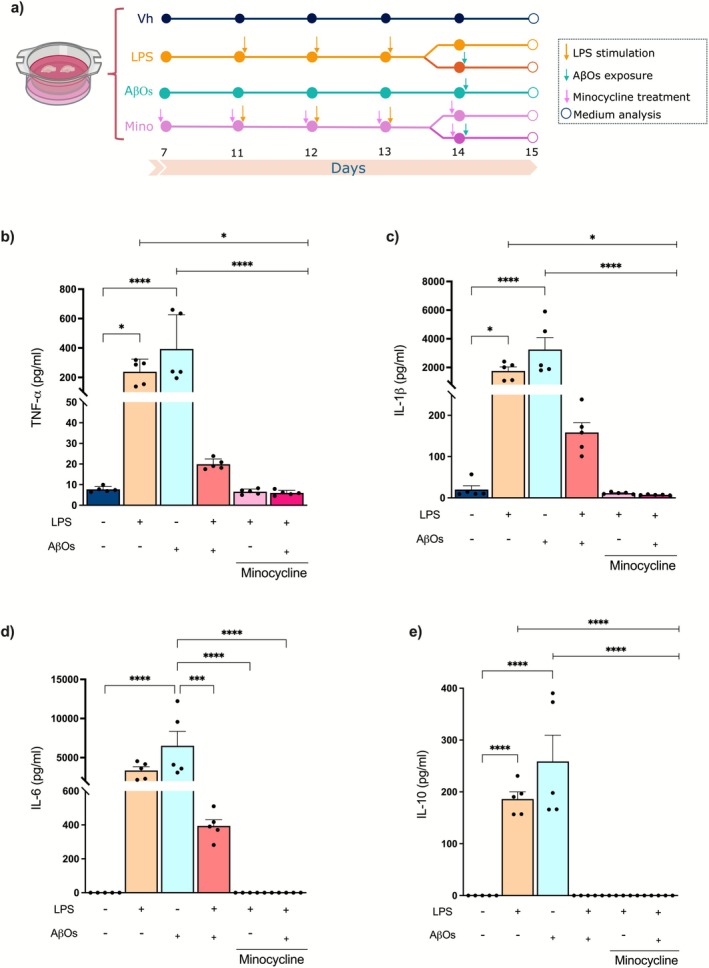
Tolerance in microglia reduces the levels of inflammation induced by AβOs. Organotypic hippocampal slice cultures were repeatedly challenged with LPS and 72 h after the first LPS stimulation exposed to AβOs. The cytokine levels were analyzed in the culture medium 24 h after AβOs exposure. (a) Schematic timeline illustrating minocycline treatment, repeated LPS stimulation, and AβOs exposure of organotypic hippocampal slice cultures. (b) TNF‐α, (c) IL‐1β, (d) IL‐6, and (e) IL‐10 levels in the culture medium 24 h after exposure of the cultures to AβOs (*n* = 5 independent cultures per group; 3 animals/culture). The results are presented as the mean ± standard error of mean (mean ± SEM). The data were analyzed using a two‐way analysis of variance (ANOVA) followed by the Holm‐Sidák test. *p < 0.05, ***p < 0.001, ****p < 0.0001.

Neuroinflammation is widely recognized as a key feature of the neurodegenerative process in AD (Castro‐Gomez and Heneka 2024; Hayek et al. 2024). Since Aβ activates microglia and increases the production of proinflammatory cytokines (Ledo et al. 2013; Ledo et al. 2016; Heneka et al. 2025), we asked whether a diminished ability of organotypic hippocampal slice cultures to produce proinflammatory cytokines following repeated challenges with LPS could mitigate the increase in these cytokines induced by AβOs. To test this, we analyzed the levels of TNF‐α, IL‐β, IL‐6, and IL‐10 in the medium after slice cultures were exposed to AβOs for 24 h, following or not previous repeated stimulation by LPS (Figure 2a). Slices exposed solely to AβOs exhibited significant increases in levels of TNF‐α [*F* (5, 24) = 13.38; *p* < 0.0001], IL‐1β [*F* (5, 24) = 14.33; *p* < 0.0001], IL‐6 [*F* (5, 24) = 12.00; *p* < 0.0001], and IL‐10 [*F* (5, 24) = 29.91; *p* < 0.0001] in the medium (Figure 2b–e). Remarkably, when the slices were pre‐exposed to repeated LPS stimulation, the increase in inflammatory cytokines induced by AβOs was prevented (Figure 2b–e). Slices in which microglial activation had been inhibited by minocycline or depleted by PLX5622 treatment did not show any increase in proinflammatory cytokines after the LPS challenge and subsequent AβOs exposure, including TNF‐α [*F* (5, 6) = 286.4; *p* < 0.0001], IL‐1β [*F* (5, 6) = 11.30; *p* = 0.0052] and IL‐6 [*F* (5, 6) = 4515; *p* = 0.0470] (Figure 2b–e, Figure S3).

To evaluate whether chronic LPS stimulation or AβOs exposure affected slice viability, we measured LDH leakage in the media collected 24 h after AβOs exposure or each LPS challenge. LDH levels did not significantly differ between LPS‐treated and untreated slices, nor between AβO‐exposed slices or those challenged with LPS followed by AβOs exposure (Figure S1). In summary, these results show that repeated stimulation of organotypic hippocampal slice cultures with LPS resulted in a decreased AβO‐induced inflammatory response.

### 3.3 Chronic Stimulation With LPS Reduces Microglial Activation Induced by AβOs

**FIGURE 3:**
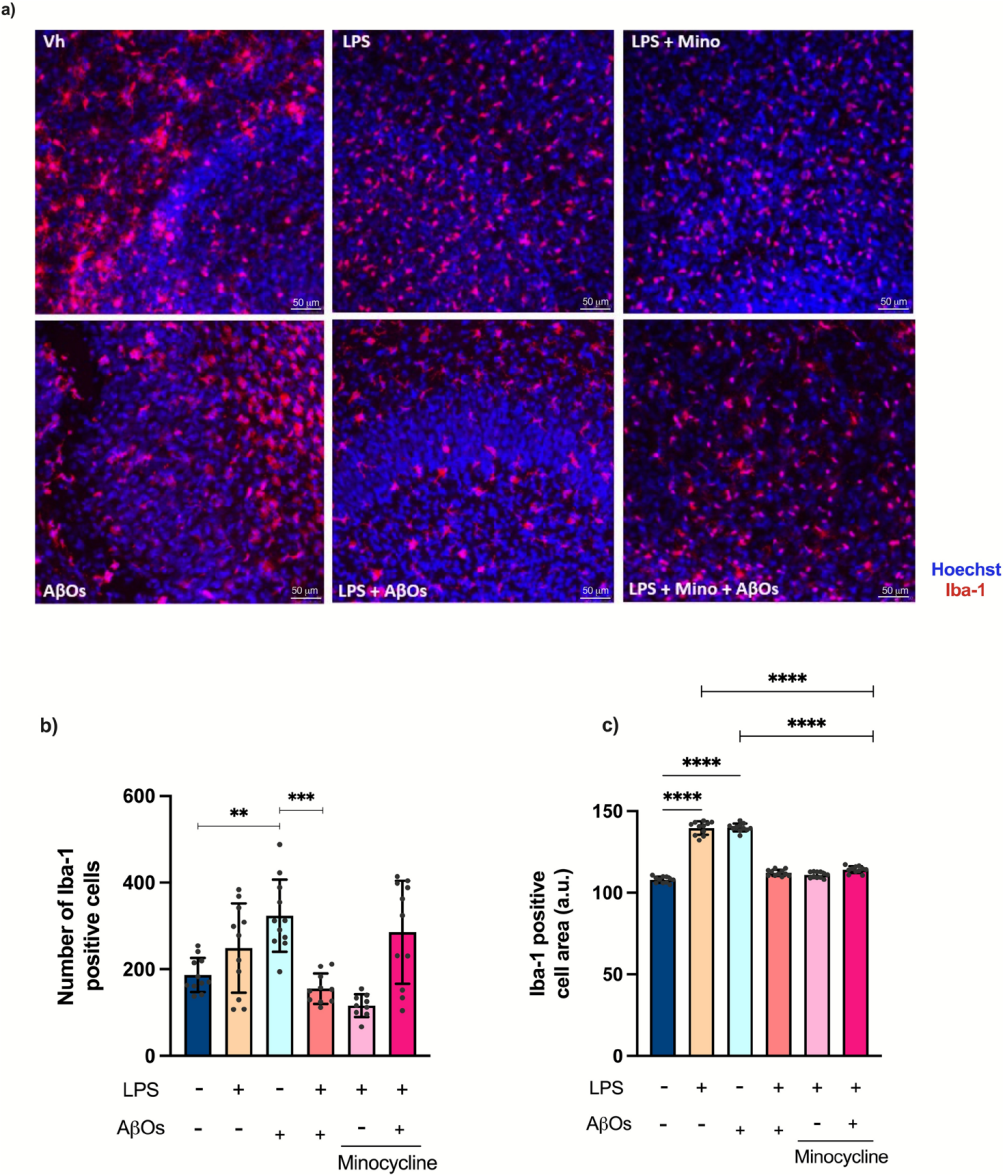
Chronic stimulation of organotypic hippocampal slice cultures with LPS decreases the AβOs‐induced microglial activation. Microglial activation was evaluated after repeated stimulation with LPS, followed by AβOs exposure. (a) Photomicrographs showing microglial cells labeled for Iba‐1 (red) and Hoescht (blue). (b) Quantification of the number of Iba‐1 positive cells. (c) Quantification of the area of Iba‐1 positive cells. Images were obtained by confocal microscopy. Scale bars: 50 μm. (*n* = 8/12 images per group). The results are presented as the mean ± standard error of mean (mean ± SEM). The data were analyzed using a two‐way analysis of variance (ANOVA) followed by the Holm‐Sidák test. **p < 0.01, ***p < 0.001, ****p < 0.0001.

To investigate whether microglial density changed due to repeated stimulation with LPS followed by AβOs exposure, we performed immunofluorescence staining for Iba‐1, a marker of activated microglia (Figure 3a). We found a higher number of Iba1‐positive cells [*F* (5, 57) = 10.63; *p* < 0.0001] (Figure 3b) and a larger area occupied by these cells in the AβOs‐exposed slices [*F* (5, 60) = 394.3; *p* < 0.0001] (Figure 3c). Chronic stimulation with LPS followed by AβOs exposure led to a significant reduction in both the number and the área of Iba‐1‐positive cells compared to cultures exposed to AβOs alone (Figure 3b,c).

Notably, inhibiting microglial activation with minocycline reduced the number of Iba‐1‐positive cells in the slices repeatedly stimulated with LPS (Figure 3b), and also decreased the area occupied by these cells in slices repeatedly stimulated with LPS and subsequently exposed to AβOs (Figure 3c). As expected, the depletion of microglia abolished the staining for Iba‐1 [*F* (5, 65) = 88.17; *p* < 0.0001] (Figure S4). These findings suggest that repeated LPS challenges lead to changes in microglial coverage and activation, consistent with the development of immune tolerance.

### 3.4 Chronic Stimulation With LPS Leads to Changes in the Morphology of Microglia

**FIGURE 4:**
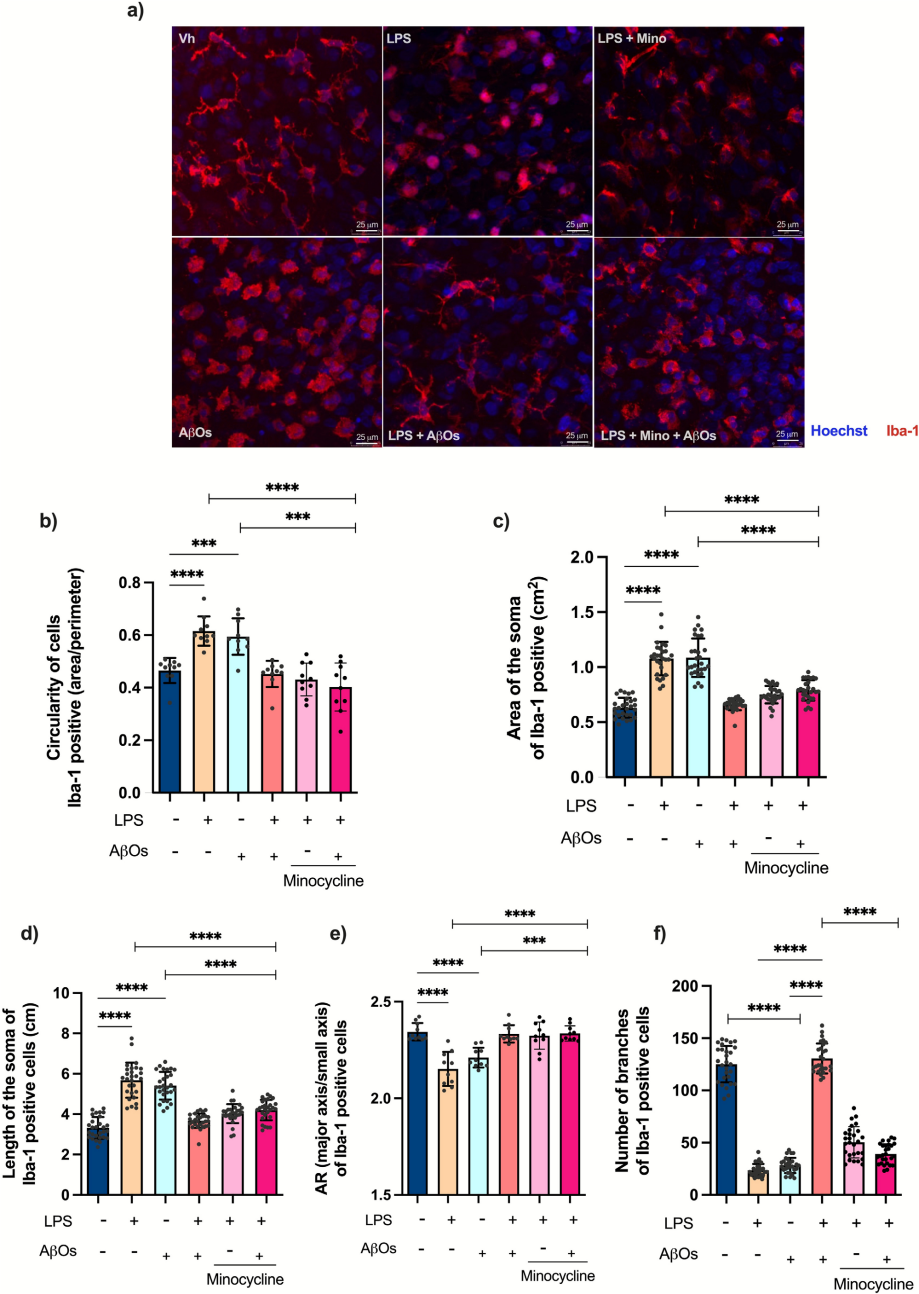
AβOs‐induced changes in the morphology of microglia are prevented by chronic stimulation with LPS. The morphology of microglia was evaluated in organotypic hippocampal slice cultures after minocycline treatment and repeated LPS challenges, followed by AβOs exposure. (a) Photomicrograph showing microglial cells stained for Iba‐1 (red) and Hoescht (blue) 24 h after AβOs exposure. Images were obtained by confocal microscopy. Scale bars: 25 μm. (*n* = 4 independent cultures per group). (b) Quantification of the circularity, (c) Quantification of the area of the soma, (d) Analysis of the length of the soma, (e) Aspect Ratio (AR) parameter levels, and (f) Number of branching junctions of Iba‐1 positive cells. (*n* = 20 cells examined per group). The results are presented as the mean ± standard error of mean (mean ± SEM). The data were analyzed using a two‐way analysis of variance (ANOVA) followed by the Holm‐Sidák test. ***p < 0.001, ****p < 0.0001.

As we observed a reduction in both the number and cell area of Iba‐1‐positive cells in hippocampal slices that were previously stimulated with LPS and exposed to AβOs, we asked whether these changes could be linked to microglial morphological alterations. We then evaluated morphological changes by assessing circularity, soma area, soma length, aspect ratio, and the number of branches in Iba‐1‐positive cells (Figure 4). The transition from more ramified toward more amoeboid shapes indicates potential changes in microglial states, characterizing an activated cell (Labzin et al. 2018; Neher and Cunningham 2019; Paolicelli et al. 2022). Exposure of organotypic hippocampal slice cultures to the AβOs resulted in increased circularity [*F* (5, 54) = 19.19; *p* < 0.0001], as well as greater soma area [*F* (5, 162) = 85.60; *p* < 0.0001] and length [*F* (5, 162) = 71.81; *p* < 0.0001] of Iba‐1‐positive cells. However, prior stimulation with LPS significantly decreased these changes (Figure 4a–d). Additionally, repeated stimulation with LPS or exposure to AβOs led to a reduction in the aspect ratio (AR) [*F* (5, 54) = 18.98; *p* < 0.0001], a measure of cell elongation (Figure 4e), as well as in the number of branches [*F* (3, 108) = 649.9; *p* < 0.0001] (Figure 4f) of Iba‐1‐positive cells when compared to slices exposed to AβOs after previous LPS challenges. Overall, these findings indicate that the morphological changes in microglia caused by repeated LPS stimulation of organotypic hippocampal slices are associated with the development of a tolerant phenotype, which attenuates microglia activation and aberrant inflammatory response triggered by AβOs. We observed that minocycline inhibited changes in the circularity, soma area, soma length, and aspect ratio (AR) of microglia induced by LPS stimulation or AβOs exposure (Figure 4b–e). However, it did not prevent the reduction in the number of branching junctions in Iba‐1‐positive cells. One possible explanation for this response to minocycline treatment could be that microglia polarize toward an anti‐inflammatory profile, leading to a reduction in cytokine secretion while still exhibiting an amoeboid morphology.

**FIGURE 5:**
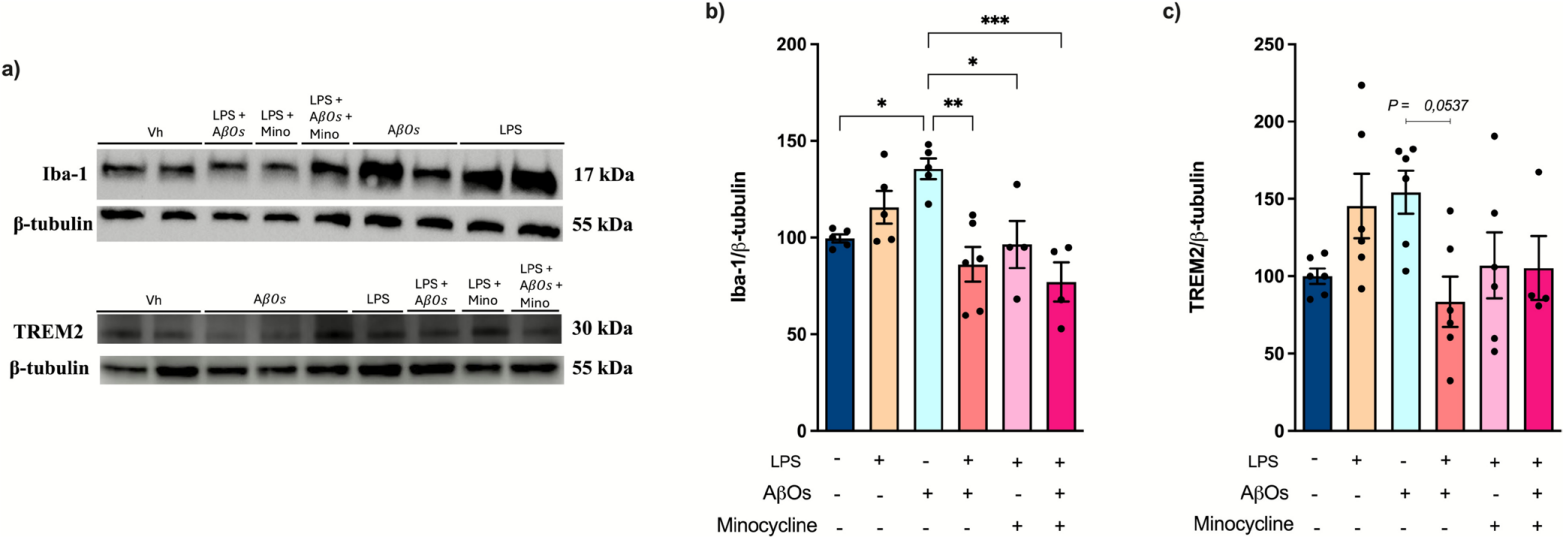
Tolerance in microglia leads to a decrease in the expression of markers associated with microglial activation. Organotypic hippocampal slice cultures were repeatedly challenged with LPS, followed by exposure to AβOs. The expression of Iba‐1 and TREM2 was analyzed by western blotting 24 h after exposure to AβOs. (a) Representative images of immunoblotting of Iba‐1, TREM2, and β‐tubulin. (b) Quantification of Iba‐1 immunocontent levels normalized by β‐tubulin. (c) Quantification of TREM2 immunocontent normalized by β‐tubulin. The y‐axis represents the percentage relative to the intensity of the immunoblotting (% of Control). (*n* = 5/7 animals per group). The results are presented as a percentage of the mean ± standard error of the mean (mean ± SEM). The data were analyzed using a two‐way analysis of variance (ANOVA) followed by the Holm‐Sidák test. *p < 0.05, **p < 0.01, ***p < 0.001.

Next, we evaluated whether challenges with LPS, exposure to AβOs, and sequential challenges with LPS followed by AβOs exposure affected the expression of Iba‐1 in organotypic hippocampal slice cultures. Western blotting showed that AβOs caused a significant increase in the Iba‐1 immunocontent [*F* (5, 23) = 6.552; *p* = 0.0006] (Figure 5a,b). Conversely, prior LPS challenges effectively reduced the Iba‐1 expression induced by AβOs (Figure 5a,b). Since the activation of triggering receptor expressed on myeloid cells 2 (TREM2), a cell surface receptor expressed in microglia and the major regulator of the transition from homeostatic microglia to disease‐associated microglia (Keren‐Shaul et al. 2017; Krasemann et al. 2017), has been linked to AD development (Jonsson et al. 2013; Guerreiro et al. 2013), we investigated whether repeated LPS stimulation of organotypic hippocampal slice cultures could change their expression. We found that both LPS challenges and AβOs exposure increased the expression of TREM2 [*F* (5, 28) = 2.772; *p* = 0.0372] (Figure 5a,c; *p* = 0.0537), whereas repeated LPS stimulation followed by AβOs exposure prevented this change (Figure 5a,c). Consistent with the reduction of inflammatory cytokines, the inhibition of microglial activation by minocycline decreased the expression of both Iba‐1 and TREM2 proteins (Figure 5a–c).

### 3.5 Chronic Stimulation With LPS Inhibits the Epigenetic Changes Caused by AβOs

**FIGURE 6:**
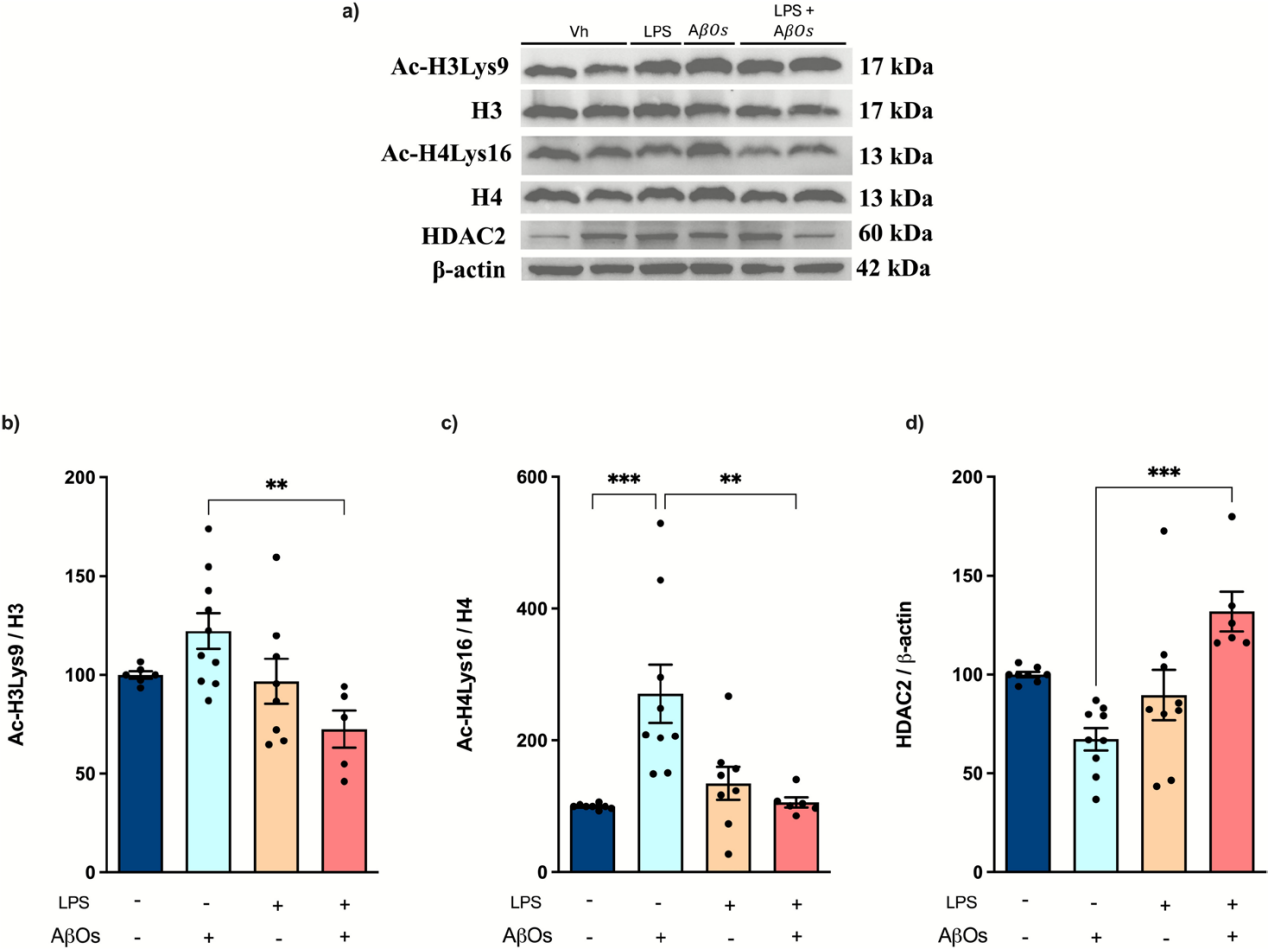
LPS‐induced innate immune tolerance leads to epigenetic modifications. Organotypic hippocampal slice cultures were repeatedly challenged with LPS, followed by exposure to AβOs. The acetylation of histones H3 and H4, as well as the expression of HDAC2, was evaluated by western blotting 24 h after exposure to AβOs (48 h after the last LPS stimulation). (a) Representative images showing the immunoblotting of histone 3 (acetyl‐Lys9 H3 and total H3), histone 4 (acetyl‐Lys16 H4 and total H4), HDAC2, and β‐actin. (b) Ratio of acetyl‐Lys9 H3 to H3. (c) Ratio of acetyl‐Lys16 H4 to H4. (d) Quantification of HDAC2 immunocontent normalized by β‐Actin. The y‐axis represents the percentage relative to the intensity of the immunoblotting (% of control). (*n* = 6–9 animals per group). The results are presented as a percentage of the mean ± standard error of the mean (mean ± SEM). The data were analyzed using a two‐way analysis of variance (ANOVA) followed by the Holm‐Sidák test. **p < 0.01; ***p < 0.001.

Once the establishment of innate immune tolerance has been linked to epigenetic changes (Scholz et al. 2024; Martins‐Ferreira et al. 2021), we aimed to determine whether repeated stimulation with LPS could inhibit histone acetylation. We found that exposure of cultures to AβOs led to increased acetylation of histones H3 [*F* (3, 25) = 4.394; *p* = 0.0129] and H4 [*F* (3, 27) = 8.254; *p* = 0.0005] (Figure 6a–c). This enhanced histone acetylation was accompanied by a significant decrease in the expression of histone deacetylase 2 (HDAC2) [*F* (3, 28) = 8.435; *p* = 0.0004] (Figure 6a,d). Although repeated stimulation with LPS does not seem to alter HDAC2 expression or the acetylation of histones H3 and H4, previous exposure to LPS followed by AβOs significantly increased HDAC2 expression and prevented the enhanced acetylation of histones (Figure 6a–d).

### 3.6 Chronic Stimulation With LPS Decreases the Activation of Inflammatory Cell Signaling Pathways Induced by AβOs

Microglial activation, characterized by an increased production of cytokines, is associated with alterations in various signaling pathways related to the inflammatory responses. Therefore, we conducted a western blot analysis to assess the expression/phosphorylation levels of proteins Jun N‐terminal kinase (JNK), p65 subunit of nuclear factor‐kappa B (p65 NF‐κB), inhibitor of nuclear factor‐kappa B (IκBα), NF‐κB subunit RelB, and cyclooxygenase‐2 (COX‐2) to determine whether LPS was influencing the development of immune tolerance by modulating these proteins.

**FIGURE 7:**
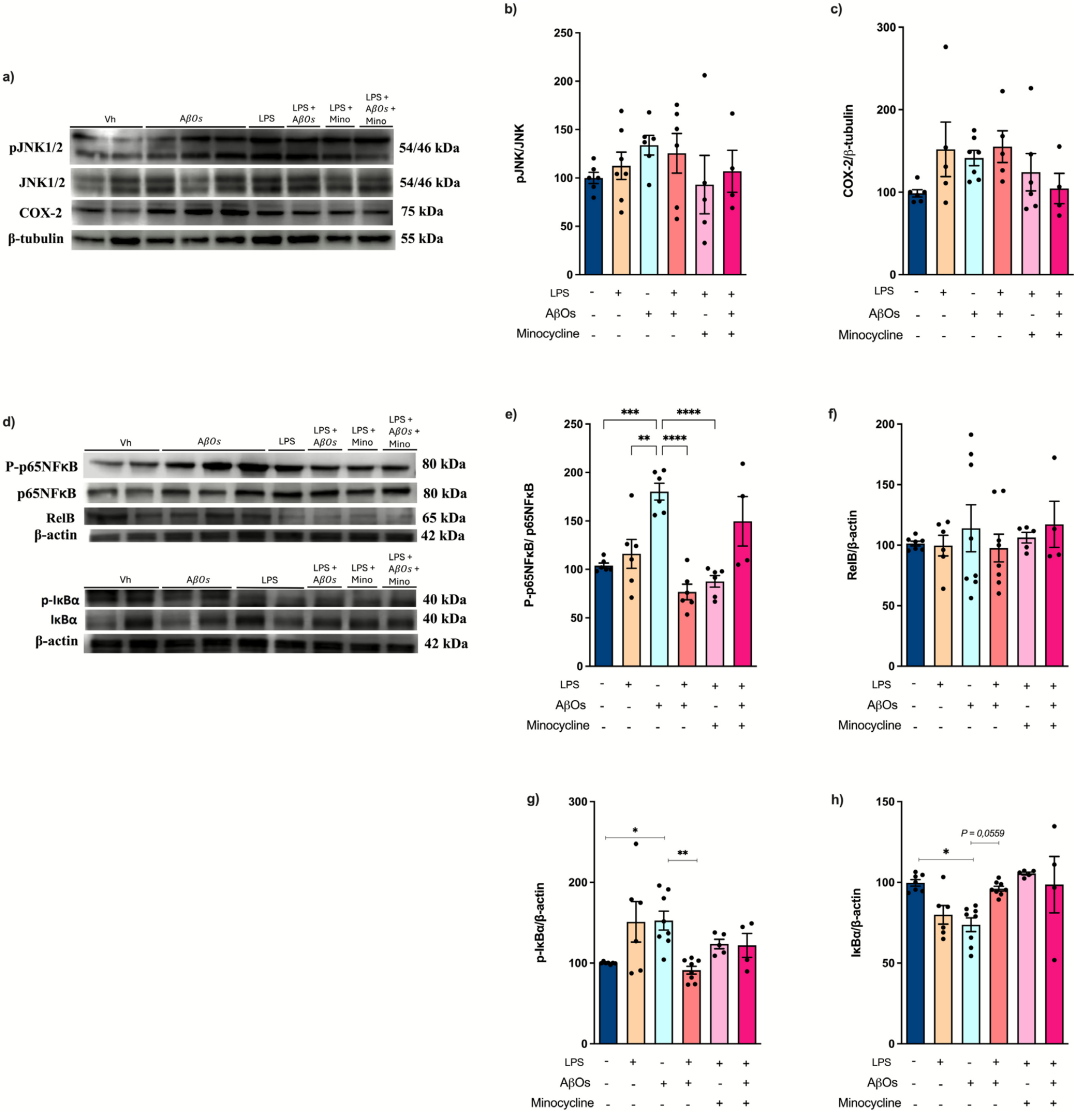
AβOs‐Induced expression of inflammatory markers is prevented by repeated stimulation with LPS. Organotypic hippocampal slice cultures were repeatedly challenged with LPS followed by exposure to AβOs. The expression of proteins associated with inflammatory cell signaling pathways was analyzed by western blotting in slices 24 h after exposure to AβOs. (a) Representative immunoblotting images of pJNK, JNK, COX‐2, and β‐tubulin. (b) Quantification of pJNK immunocontent normalized by JNK. (c) Quantification of COX‐2 immunocontent normalized by β‐tubulin. (d) Representative immunoblotting images of P‐p65NF‐κB, p65NF‐κB, RelB, p‐IκBα, IκBα, and β‐actin. (e) Quantification of P‐p65NF‐κB expression normalized by p65NF‐κB. (f) Quantification of RelB immunocontent normalized by β‐Actin. (g) Quantification of p‐IκBα immunocontent normalized by β‐actin. (h) Quantification of IκBα immunocontent, normalized by β‐Actin. The *y*‐axis represents the percentage relative to the intensity of immunoblotting (% of control). (*n* = 5/7 animals per group). The results are presented as the mean ± standard error of mean (mean ± SEM). The data were analyzed using a two‐way analysis of variance (ANOVA) followed by the Holm‐Sidák test. *p < 0.05, **p < 0.01, ***p < 0.001, ****p < 0.001.

All slices exhibited comparable levels of JNK phosphorylation [*F* (5, 46) = 1.125; *p* = 0.3606] (Figure 7a,b) and RelB expression [*F* (5, 32) = 0.3488; *p* = 0.8792] (Figure 7d,f). Although not statistically significant, both LPS‐stimulated and AβOs‐exposed slices displayed increased COX‐2 expression [*F* (5, 27) = 1.558; *p* = 0.2054] (Figure 7a,c). Similar increases in COX‐2 expression were observed in slices previously challenged with LPS and then exposed to AβOs. While LPS stimulation did not affect p65 NF‐κB phosphorylation levels, exposure to AβOs resulted in increased phosphorylation of p65 NF‐κB. Notably, this increase was blocked in slices that had previously been challenged with LPS [*F* (5, 28) = 12.42; *p* < 0.0001] (Figure 7d,e). In addition, both LPS and AβOs led to elevated levels of phosphorylated IκBα protein. However, slices that were first stimulated with LPS and then exposed to AβOs did not show changes in the IκBα phosphorylation [*F* (5, 32) = 4.831; *p* = 0.0021] (Figure 7d,g). Consistent with the activation of the NF‐κB pathway, the expression of IκBα was reduced following LPS stimulation or AβOs exposure (Figure 7d,h). In contrast, slices exposed to AβOs after LPS stimulation exhibited IκBα expression similar to that of control slices [*F* (5, 32) = 5.484; *p* = 0.0009] (Figure 7d,h; *p* = 0.0559). Inhibition of microglial activation with minocycline prevented the increase in IκBα phosphorylation (Figure 7d,g), as well as COX‐2 expression (Figure 7a,c) and the reduction in IκBα (Figure 7d,h). However, minocycline treatment did not affect RelB expression levels (Figure 7d,f). Furthermore, microglial inhibition did not prevent the increased phosphorylation of p65 NF‐κB induced by LPS stimulation followed by AβOs exposure (Figure 7d,e). In summary, this data supports the role of the NF‐κB pathway in the innate immune tolerance induced by LPS.

### 3.7 Chronic Stimulation With LPS Prevents Changes in the Expression of Synaptic Proteins and GFAP Induced by AβOs

In addition to epigenetic changes and the activation of pro‐inflammatory signaling pathways, we evaluated the effects of microglial tolerance on synaptic plasticity by examining the expression of synaptophysin and PSD‐95 (postsynaptic density protein 95), which are presynaptic and postsynaptic proteins, respectively. Our analysis revealed that slices exposed solely to AβOs exhibited a significant increase in PSD‐95 levels [*F* (5, 29) = 6.566; *p* = 0.0003] (Figure S5a). In contrast, no significant change was observed in the expression of synaptophysin [*F* (5, 27) = 6.926; *p* = 0.0005] (Figure S5a,b). Furthermore, slices that were first stimulated with LPS and then exposed to AβOs showed a significant reduction in both synaptophysin and PSD‐95 compared to the slices exposed only to AβOs (Figure S5a,b,c). Notably, treatment with minocycline did not alter the expression of either PSD‐95 or synaptophysin, with levels remaining similar to those of the control slices (Figure S5a,b,c).

Research has shown that both neurons and astrocytes are essential for inducing tolerance in microglia (Chu et al. 2016). To explore this further, we examined whether repeated LPS stimulation of organotypic hippocampal slice cultures affected astrocyte activation. We found that expression of GFAP, a marker of astrocytic reactivity, increased in slices exposed solely to AβOs [*F* (5, 25) = 3.415; *p* = 0.0173] (Figure S5). In contrast, repeated LPS stimulation resulted in only a slight increase in GFAP expression. Interestingly, when slices were stimulated with LPS and subsequently exposed to AβOs, the increase in GFAP expression caused by AβOs was blocked (Figure S5).

## 4 Discussion

Microglia play crucial roles in a healthy CNS, and changes in their activity have been linked to the development of neurodegenerative diseases (Sierra et al. 2024). Additionally, it has been demonstrated that microglia adapt their responses to inflammatory events based on prior exposure to stimuli (Neher and Cunningham 2019; Wendeln et al. 2018; Zhang et al. 2022; Kuo et al. 2023; Meng et al. 2024; Kim et al. 2024), bringing the notion of innate immune memory (Divangahi et al. 2021). A large body of evidence indicates that primed or trained microglia display an exaggerated immune response, characterized by increased production of pro‐inflammatory cytokines (Cunningham et al. 2005; Sierra et al. 2007; Wendeln et al. 2018; Heng et al. 2021; He et al. 2024). This acquired immune memory may make microglia more sensitive to various immune stimuli, such as aggregated proteins that accumulate in neurodegenerative disorders (Norden et al. 2015; Frost et al. 2019). Conversely, microglia can also exhibit immune tolerance, resulting in a diminished immune response following subsequent challenges (Schaafsma et al. 2015; Yu et al. 2010; Ye et al. 2024; Wendeln et al. 2018), potentially preventing or mitigating neurodegeneration.

In the current study, we evaluated the development of innate immune tolerance in microglia and its influence on the subsequent inflammatory response caused by AβOs, proximal neurotoxins involved in AD (Ferreira and Klein 2011; Frisoni et al. 2022). We found that LPS significantly increased the contents of TNF‐α, IL‐1β, and IL‐6 from undetectable levels in the control slices to very high levels during the first 24 h following the LPS challenge. Interestingly, the increases in cytokine levels were gradually dampened following subsequent LPS challenges. This dampening is consistent with previous research reporting microglial polarization toward an anti‐inflammatory profile following persistent LPS stimulation (Antonietta Ajmone‐Cat et al. 2013).

A similar pattern was observed for the anti‐inflammatory cytokine IL‐10, although its reduction after subsequent LPS challenges was less pronounced. It is important to note that most of our current knowledge on immune training and tolerance comes from studies on cultured peripheral macrophages. However, neurons and astrocytes appear to be indispensable for developing tolerance in microglia (Chu et al. 2016). To minimize potential interference in developing microglial tolerance due to the absence of their interaction with neural cells, we took advantage of organotypic hippocampal slice cultures as an intermediate model between classic cell cultures and in vivo models, preserving tissue structure while maintaining neuronal activity, synaptic circuitry, and glia–neuron interactions. Additionally, we used minocycline to selectively inhibit microglia and investigate whether the increased cytokine levels were mediated by these cells, since it has been suggested that long‐lived non‐immune cells might also carry innate immune memory (Hamada et al. 2019). Minocycline treatment effectively prevented the rise of TNF‐α, IL‐1β, IL‐6, and IL‐10 levels induced by the LPS challenge. Overall, our results indicate that the reduced responsiveness of microglia to repeated LPS challenges, evidenced by lower production of inflammatory cytokines, corresponds to the development of endotoxin tolerance, a form of innate immune memory. These findings are consistent with earlier studies reporting that increasing concentrations of LPS in neuron–glia cultures led to a decrease in TNF‐α levels (Chu et al. 2016), while repeated LPS preconditioning in BV2 microglial cells resulted in decreased mRNA levels of IL‐1β, IL‐6, and TNFα after subsequent high‐dose LPS challenge (Dong et al. 2024).

Neuroinflammation is increasingly recognized as a crucial factor in the pathology of AD (Wilson et al. 2023). In response to Aβ accumulation, microglia become activated and release proinflammatory cytokines, resulting in a chronic state of neuroinflammation (Hansen et al. 2018). This persistent inflammation contributes to disease progression. Consistent with this evidence, we showed that AβOs led to increased levels of proinflammatory cytokines TNF‐α, IL‐1β, and IL‐6 in organotypic hippocampal slices. In addition to the increase in cytokine levels, AβOs exposure resulted in heightened expression of Iba‐1 and a greater number of Iba‐1‐positive cells with larger cell areas, indicating microglial activation. On the other hand, modeling innate immune tolerance in microglia of organotypic hippocampal slice cultures, we found that repeated challenges with LPS led to a reduction in microglial activation and the inflammatory response caused by the AβOs.

Although previous studies have shown that microglial preconditioning with LPS results in changes in their function (Heng et al. 2021; Kim et al. 2024; Zhang et al. 2022), published experiments were centered on the evaluation of how microglia can establish trained or tolerance immune memory. By contrast, there is a scarcity of studies showing how preconditioned microglia react to secondary inflammatory stimuli that contribute to the development of neurodegenerative disorders. A seminal study by Wendeln and colleagues provided the first evidence of the impact of both immune training and tolerance in microglia on the neuropathology of AD and stroke (Wendeln et al. 2018). Similarly, Yang and collaborators showed that priming microglia before plaque deposition inhibited their activation and limited Aβ pathology (Yang et al. 2023). These studies utilized transgenic animals that produce Aβ early and involved peripheral LPS administration, which does not rule out the possible influence of peripheral innate and adaptive immune cells upon findings in the brain. By evaluating the effect of LPS preconditioning in organotypic hippocampal slice cultures, where microglia are the unique immune cell, our findings support previous data showing that innate immune memory can yield protection against both homo‐ and heterologous secondary stimuli (Netea et al. 2020).

Microglial cells respond to challenges in their microenvironment by altering their molecular profile, morphology, and ultrastructure, as well as motility and function (Paolicelli et al. 2022). We found that exposure to AβOs led to increased circularity and length of soma while reducing the aspect ratio and the number of branches of Iba‐1 positive cells. These changes, along with elevated cytokine levels, indicate microglial activation. Conversely, repeated challenges with LPS before AβOs exposure effectively prevented these alterations. Although there is ongoing discussion regarding the definition of microglial activation and its inflammatory responses linked to specific morphological changes (Paolicelli et al. 2022), the increased levels of IL‐1β associated with reduced microglial branching are in line with the previous findings demonstrating a mechanistic link between microglial morphology changes and inflammatory responses (Madry et al. 2018). We observed that AβOs not only induced changes in the morphology of microglia but also increased the expression of Iba‐1 and TREM2. Since TREM2 plays a relevant role in microglial function during the development of AD (Guerreiro et al. 2013; Ulland and Colonna 2018), our findings suggest that modulating microglial immunological memory may be a promising strategy for controlling their function. Yet, single‐cell, multi‐omics, and integrative analyses of gene and protein expression will provide further insight into determining specific cell states.

Long‐lasting epigenetic modifications and metabolic reprogramming characterize innate immune memory (Netea et al. 2020; Saeed et al. 2014; Lajqi et al. 2019; Zhang et al. 2022; Novakovic et al. 2016). Consistent with this notion, our study revealed that exposing cultures to AβOs significantly increased the acetylation of histones H3 and H4, while also leading to a notable decrease in HDAC2 expression. This suggests that the epigenetic environment became more permissive for gene transcription. This observation aligns with existing literature, which shows that AβOs are associated with changes in the acetylation of histone H3 (including H3K14ac, H3K9ac, H3K27ac) and histone H4 (specifically H4K5ac and H4K12ac) in chromatin of pro‐inflammatory genes (Fisher and Torrente 2024; Basheer et al. 2024). The increased acetylation likely enhances gene accessibility and primes cells for an inflammatory response induced by AβOs. On the other hand, in our experiments, we observed a reduction in the acetylation levels of histones H3 and H4, alongside an increase in HDAC2 expression in cultures repeatedly stimulated with LPS and exposed to AβOs. This reduction in acetylation may indicate a transcriptionally repressive state, as demonstrated in previous studies (Wendeln et al. 2018; Scholz et al. 2024). Furthermore, the increased expression of HDAC2 suggests that histone deacetylases could serve as central mediators of epigenetic reprogramming of microglia during tolerance. These findings highlight the potential role of epigenetic modifications in the development of microglial tolerance. Although preliminary, these results pave the way for further investigations into epigenetic modifications with metabolic reprogramming in tolerant microglia, impacting neurodegeneration associated with AD.

In addition to epigenetic changes, signaling pathways related to inflammation may also play a role in this immune response, as it has been demonstrated that p38 and ERK1/2 mediate the establishment of innate immune memory (Kuo et al. 2023; Lajqi et al. 2019). We found an elevation in the expression of COX‐2 in response to both LPS challenge and AβOs. Although repeated LPS stimulation before AβOs exposure did not prevent the increase of COX‐2 expression, our result corroborates a previous study showing that the elevated expression of COX‐2 is sustained in Iba‐1‐positive cells (Antonietta Ajmone‐Cat et al. 2013).

Another key regulator of inflammatory cell signaling that appears to be central to the modulation of innate immune memory and the inflammation triggered by AβOs is the NF‐κB pathway (Valerio et al. 2006; Schaafsma et al. 2015; Seeley and Ghosh 2017; Kim et al. 2024; Ju Hwang et al. 2019; Sun et al. 2022). In line with this evidence, we found that AβOs induced increased phosphorylation of IκB and a reduction in its expression. This suggests that IκB is being targeted for degradation, which allows the p65 NF‐κB subunit to be released (Wang et al. 2020). The release of p65, followed by an increase in its phosphorylation induced by AβOs, indicates activation of the NF‐κB pathway, resulting in elevated production of cytokines (Sun et al. 2022; Lindsay et al. 2021). Notably, these changes were prevented by prior challenges with LPS even in the presence of AβOs. In contrast with a previous study demonstrating that suppression of the pro‐inflammatory response of microglia induced by LPS‐preconditioning is mediated by RelB‐dependent epigenetic silencing, we did not observe any changes in the phosphorylation of RelB (Schaafsma et al. 2015). Furthermore, we observed changes in the expression of both phosphorylated and total levels of IκBα only in the slices that experienced repeated LPS stimulation, while no changes were noted in the phosphorylation of p65 NF‐κB.

Typically, the inflammatory response triggered by LPS is associated with the activation of the NF‐κB pathway. However, it is important to note that the binding of pathogen‐associated molecular patterns (PAMPs), such as LPS, to toll‐like receptor 4 (TLR4) can initiate a signaling cascade involving both NF‐κB‐dependent and NF‐κB‐independent pathways (Pålsson‐McDermott and O'Neill 2004). There may be a negative regulatory relationship between these two pathways, influenced by various molecules or signaling mechanisms. For instance, in macrophages, LPS can inhibit NF‐κB signaling by inducing the expression of Inducible cAMP early repressor (ICER) through a p38‐mediated pathway that activates cAMP response element‐binding protein (CREB). This process establishes a negative feedback loop, which is crucial for preventing excessive inflammation driven by TLR activation (Lv et al. 2017). The downstream kinase mitogen‐and stress‐activated protein kinase (MSK)1/2 of p38 phosphorylates and activates the transcription factor CREB, which promotes the transcription of related genes (Ananieva et al. 2008). Phosphorylated CREB can inhibit NF‐κB activation by competing with p65 for binding to CREB‐binding protein (CBP) (Parry and Mackman 1997). Additionally, it has been demonstrated that once LPS is internalized by target cells, it does not lead to the translocation of NF‐κB or the transcription of cytokine mRNA, nor does it induce NF‐κB‐dependent protein expression (Seitzer and Gerdes 2003). Similarly, studies have demonstrated that the phosphorylation of IκBα and its subsequent degradation do not occur in LPS‐tolerant cells (Fujihara et al. 2000). Finally, it has been reported that low concentrations of LPS fail to induce the degradation of IκBα or the nuclear translocation of p65/RelA, which are canonical components of the NF‐κB pathway (Maitra et al. 2012; Maitra et al. 2011). Therefore, our results suggest that repeated stimulation with low concentrations of LPS not only fails to activate the NF‐κB pathway but may also prevent its activation in the presence of AβOs.

Previous studies have shown that LPS‐induced immune training or tolerance in microglia plays a crucial role in influencing brain pathology (Wendeln et al. 2018; Dong et al. 2024; Frost et al. 2019; Charoensaensuk et al. 2024; Yang et al. 2023). However, it is important to note that innate immune memory in these studies was induced by administering LPS peripherally, and LPS is largely unable to penetrate the blood–brain barrier in healthy animals (Banks et al. 2015), even though it can trigger a neuroinflammatory response. The effects of peripherally administered LPS are likely transmitted to the brain's parenchyma via TLR4 receptors on meningeal and endothelial cells, which in turn signal to other brain cell types, including microglia. Additionally, recent findings demonstrated that innate immune memory may be driven by increased production of IL‐1β (Simats et al. 2024). Consequently, the changes in microglia function could be influenced by cytokines produced by peripheral immune cells. Overall, our findings reveal, for the first time, that repeated LPS stimulation directly affects microglia, shifting their functions toward an anti‐inflammatory profile that can mitigate the heightened inflammatory response triggered by AβOs.

Since both LPS and AβOs can signal through TLR4, a reduction in TLR4 expression resulting from repeated LPS stimulation could decrease the inflammatory response triggered by AβOs. Although we did not measure TLR4 expression levels after LPS stimulation in our study, prior research has shown that AβOs increase TLR4 expression (Calvo‐Rodríguez et al. 2017). Therefore, this increase could neutralize any potential reduction in TLR4 expression caused by repeated LPS stimulation. Furthermore, pretreatment with low doses of TLR4 agonists has been shown to stimulate microglia to produce neuroprotective cytokines, including IFN‐β, which could be considered a potential strategy to combat neuronal degeneration in AD (Yousefi et al. 2019). Additionally, preliminary results indicate that microglia exhibit tolerance even after being stimulated with the cytokine IL‐1β in organotypic hippocampal slice cultures, which mimics sterile inflammation. Repeated stimulation of cultures with IL‐1β, followed by exposure to AβOs, results in decreased cytokine levels, similar to what is observed with repeated LPS stimulation (data not shown). Since IL‐1β interacts with the type 1 interleukin‐1 receptor (IL‐1R1), the mechanism behind the induction of innate immune tolerance in this context does not involve the TLR4 pathway. Therefore, our findings suggest that the reduced inflammatory response caused by AβOs after LPS stimulation does not depend on TLR4 activation.

In addition to microglia, astrocytes are essential for maintaining brain homeostasis and responding to inflammatory stimuli. Further, activated neuroinflammatory microglia can lead to increased astrocyte reactivity, which contributes to an altered inflammatory environment and results in the death of neurons and oligodendrocytes in neurodegenerative diseases (Liddelow et al. 2017; Takei et al. 2023). Consistent with previous research demonstrating that astrocytes also undergo molecular changes associated with the development of endotoxin tolerance (Chistyakov et al. 2019) and epigenetic reprogramming that enhances pro‐inflammatory responses upon subsequent challenges (Lee et al. 2024), we found that repeated stimulation of cultures to LPS prior to AβOs exposure prevented the increase in GFAP expression. This finding suggests that astrocytes may also play a role in innate immune memory. It has been recognized that activated microglia can induce neurotoxic reactive astrocytes (Liddelow et al. 2017). Therefore, it is important to determine whether reactive astrocytes are a direct response to LPS or whether they are induced by activated microglia. Notably, treatment of the cultures with minocycline inhibited the increase in GFAP expression, indicating a potential interaction between these glial cells. Future research will be needed to clarify these relationships.

In an attempt to evaluate whether microglia tolerance affects synaptic plasticity, we assessed the expression of pre‐ and post‐synaptic proteins, specifically synaptophysin and PSD95. Surprisingly, we found that both LPS stimulation and exposure to AβOs increased the expression of synaptophysin and PSD95. Although these findings may appear contradictory, they suggest increased synaptic activity following LPS or AβOs exposure, potentially linked to increased neuronal excitability, which is a phenomenon observed during neuroexcitotoxicity responses. Previous studies have indicated that Aβ leads to elevated synaptophysin expression in the cortex of transgenic mouse models of AD (Dodart et al. 2000). Furthermore, several studies have demonstrated that PSD95 is overexpressed in the early stages of the disease in patients, as well as in the hippocampus of mice injected with Aβ and in neuronal cultures exposed to AβOs (Rezaie et al. 2005; Leuba et al. 2008; Ortiz‐Sanz et al. 2022). Therefore, the increased expression of synaptophysin and PSD95 may indicate that AβOs cause a transient rise in synaptic markers due to greater neurotransmitter release into the synaptic cleft, which subsequently culminates in synaptic dysfunction and reduced expression, contributing to mild cognitive impairment in the early stages of AD (Baazaoui and Iqbal 2017). On the other hand, our results indicate that repeated LPS stimulation prevented changes in PSD95 expression and even resulted in a decrease in AβO‐induced synaptophysin expression. In the future, we aim to conduct a more comprehensive assessment of how microglial tolerance affects neuronal function.

Although this study underscores the potential of innate immune modulation in reducing the inflammatory process in a preclinical model of AD, there are several limitations. First, the activation of microglia plays a crucial role in preventing Aβ accumulation during AD pathogenesis, and determining the optimal timing for altering their activation and function is challenging. Second, the duration of innate immune memory in microglia is still largely unexplored, which raises concerns that inducing tolerant microglia over an extended period may hinder their ability to surveil the brain and protect against infections or damage. Third, while our findings demonstrate morphological changes in microglia and reduced cytokine release following repeated LPS challenges, single‐cell sequencing and spatial transcriptomics could provide deeper insights into the mechanisms underlying microglial functions in a more comprehensive manner. Additionally, our study is based exclusively on organotypic hippocampal cultures, which, while valuable, do not fully replicate the complexity of neuroinflammation observed in vivo. Given that systemic exposure to LPS has been shown to induce long‐lasting neuroimmune changes in in vivo models, investigating the effects of microglial tolerance in an animal model may enhance the translational relevance of our findings. Finally, increasing evidence suggests that peripheral infections may contribute to the pathogenesis of AD, exacerbating the condition in patients and accelerating cognitive decline. Addressing these critical issues in future studies may enhance our understanding of the role of microglial tolerance in AD and explore the broader therapeutic potential of innate immune memory in clinical treatments.

## 5 Conclusion

In conclusion, our study demonstrates that preconditioning microglia with LPS triggers physiological immune responses rather than pathological inflammation. Furthermore, the immune tolerance in microglia diminishes the inflammation caused by subsequent exposure to AβOs. This suggests that immune memory in the brain could potentially influence the severity of neurodegenerative disorders that present with an inflammatory component. These findings open new avenues for developing therapeutic strategies for AD aimed at modulating innate immune memory.

## Author Contributions

**Rafaela Rodrigues Valerio:** conceptualization, investigation, formal analysis, methodology, data curation, writing – original draft. **Áquila Rodrigues Santos:** investigation, methodology, data curation, writing – review and editing. **Ana Helena Larangeira Nóbrega:** investigation, methodology, writing – review and editing. **Raquel Martins:** methodology, validation, visualization, resources. **Fernanda G. De Felice:** writing – review and editing, data curation, funding acquisition. **Sergio T. Ferreira:** writing – review and editing, funding acquisition, formal analysis. **Wilson Savino:** writing – review and editing, funding acquisition, formal analysis. **Adriana Bonomo:** funding acquisition, writing – review and editing, formal analysis. **Andressa Bernardi:** investigation, funding acquisition, writing – review and editing, formal analysis. **Rudimar Luiz Frozza:** conceptualization, funding acquisition, visualization, formal analysis, data curation, supervision, project administration, writing – original draft, writing – review and editing.

## Funding

This work was funded by Fundação Carlos Chagas Filho de Amparo à Pesquisa do Estado do Rio de Janeiro (FAPERJ; to A.B and R.L.F.; E‐26/203.195/2016; E‐26/202.807/2019, E‐26/201.419/2021 and E‐26/200.169/2023), the Rio Network of Innovation in Nanosystems for the Health—Nanohealth/FAPERJ (E‐26/010.000983/2019), the Rio de Janeiro Network on Neuroinflammation/FAPERJ (E‐26/010.002418/2019), Inova Fiocruz Program (VPPCB‐008‐FIO‐18‐2‐52), National Institute for Translational Neuroscience (S.T. and F.G.D.F.), Conselho Nacional de Desenvolvimento Científico e Tecnológico (CNPq; 403742/2016‐1), Program Coordenação de Aperfeiçoamento de Pessoal de Nível Superior (CAPES), National Institute of Science and Technology on Neuroimmunomodulation (INCT‐NIM), and the Mercosur Program for Structural Convergence (FOCEM).

## Ethics Statement

All procedures used in the present study were performed according to the recommendations of the Guide for the Care and Use of Laboratory Animals of the National Council for Animal Experimentation and approved by the Animal Use Ethics Committee of Oswaldo Cruz Institute/Fiocruz (license L‐018/2020‐A1).

## Conflicts of Interest

The authors declare no conflicts of interest.

## Supporting information


**Data S1:** jnc70341‐sup‐0001‐Supinfo.pdf.

## Data Availability

All datasets presented in this paper are available from the corresponding authors upon reasonable request (email: rudimar.frozza@ioc.fiocruz.br).
