# Supplementary material for: Innate Immune Tolerance Regulates Microglia Response to Aβ Oligomers
Source: J Neurochem. 2026 Jan 2;170(1):e70341. doi: 10.1111/jnc.70341 (PMC12758097; doi:10.1111/jnc.70341)
Supplement: Supplementary file 1 — Data S1: jnc70341‐sup‐0001‐Supinfo.pdf. [file JNC-170-0-s001.pdf]

Innate immune tolerance regulates microglia response to Aβ oligomers

Rafaela Rodrigues Valerio[1]; Áquila Rodrigues Santos[1]; Ana Helena Larangeira Nóbrega[2], Raquel Martins[1]; Fernanda G. De Felice[3,5,6,7], Sergio T. Ferreira[3,4,6,7], Wilson Savino[1,8,9,11]; Adriana Bonomo[1,8,9,11]; Andressa Bernardi[2,9,10]; Rudimar Luiz Frozza[1,8,9,10,11*]

[1]Laboratory on Thymus Research, Oswaldo Cruz Institute, Oswaldo Cruz Foundation, FIOCRUZ, Rio de Janeiro, Brazil.

[2]Laboratory of Inflammation, Oswaldo Cruz Institute, Oswaldo Cruz Foundation, FIOCRUZ, Rio de Janeiro, Brazil.

[3]Institute of Medical Biochemistry Leopoldo de Meis and [4]Institute of Biophysics Carlos Chagas Filho, Federal University of Rio de Janeiro, Rio de Janeiro, RJ, Brazil.

[5]Centre for Neuroscience Studies, Department of Biomedical and Molecular Sciences and Department of Psychiatry, Queen's University, Kingston, ON, Canada.

[6]D'Or Institute for Research and Education, Rio de Janeiro, RJ, Brazil.

[7]National Institute of Science and Technology for Translational Neuroscience.

[8]National Institute of Science and Technology on Neuroimmunomodulation.

[9]Rio de Janeiro Research Network on Neuroinflammation/FAPERJ.

[10]Rio Network of Innovation in Nanosystems for Health - Nanohealth/FAPERJ.

[11]INOVA-IOC Network on Neuroimmunomodulation.

Contact

*Correspondence: rudimar.frozza@ioc.fiocruz.br

Supplementary Figure 1

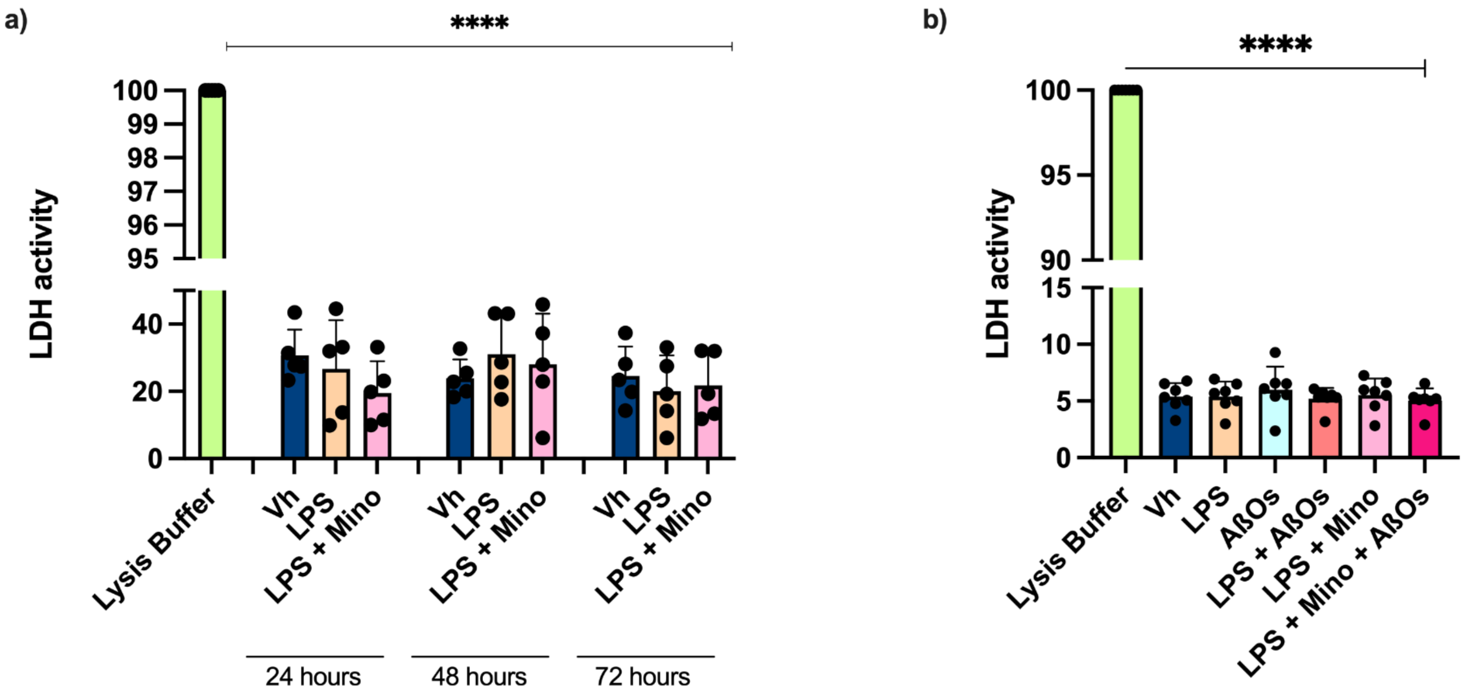

Supplementary Figure 1. Effects of repeated stimulation with LPS and AβOs exposure on cell viability. Organotypic hippocampal slice cultures were repeatedly challenged with LPS (5 µg/ml) from day 11 to day 14. The culture medium was collected and replaced every 24 h. Seventy-two hours after the first LPS stimulation, the cultures were exposed to AβOs for an additional 24 h. (a) LDH activity in the culture medium was analyzed every 24 h after each stimulation with LPS. (b) LDH activity in the culture medium was analyzed 24 h after the exposure to AβOs and 72 h after the first LPS challenge. Data are expressed as a percentage compared to the maximum LDH activity control (culture exposed to lysis buffer for 45 min), considered 100% of death. (n= 7 independent cultures per group; 3 animals/culture). The results are presented as the Mean ± Standard Error of Mean (Mean ± SEM). The data were analyzed using a two-way analysis of variance (ANOVA) followed by the Holm-Sidák test. ****$p<0.0001$.

Supplementary Figure 2

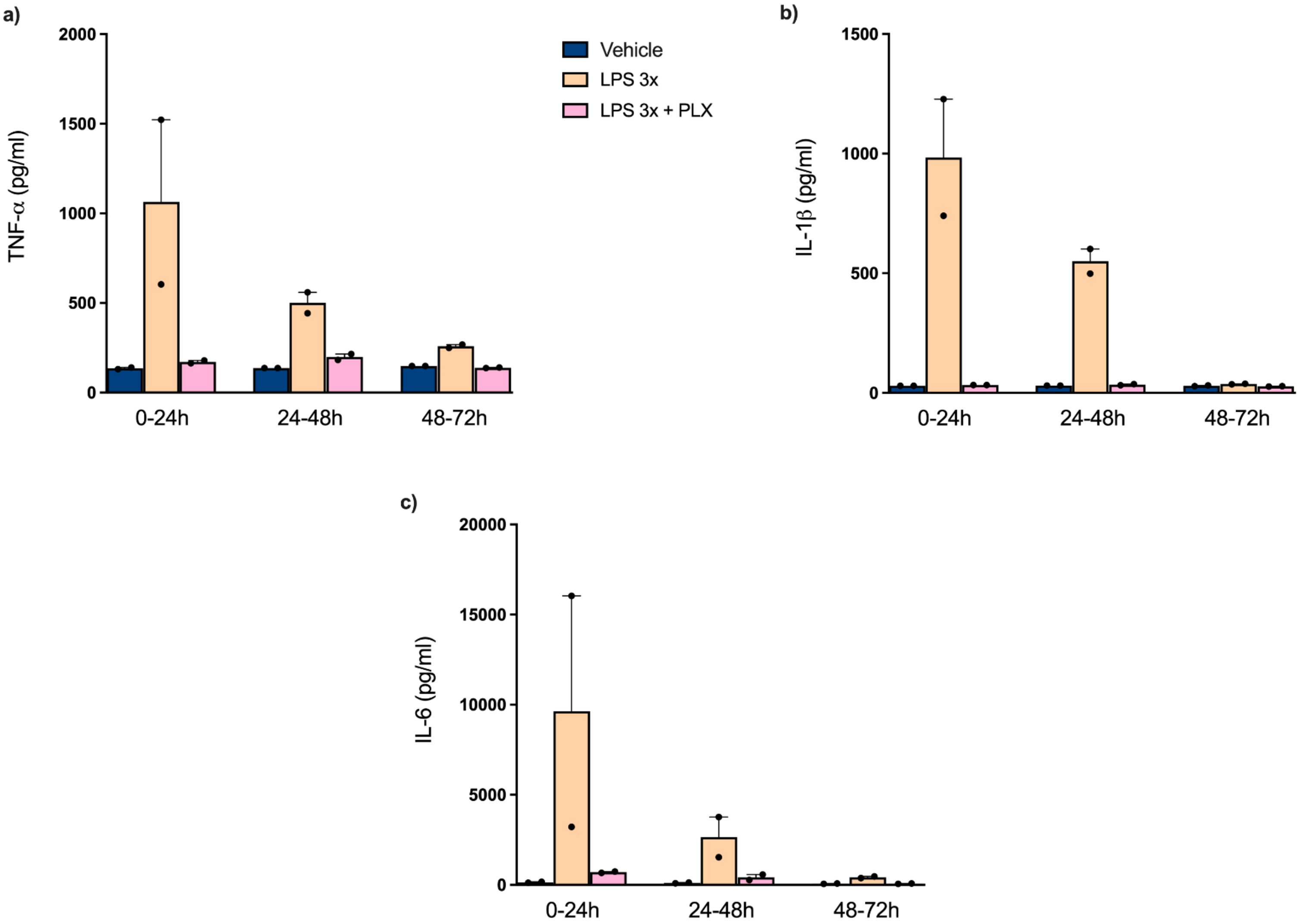

Supplementary Figure 2. Depleting microglia eliminates the elevated levels of inflammatory cytokines triggered by repeated LPS challenges in organotypic hippocampal slice cultures. Organotypic hippocampal slice cultures were cultured in the presence of 5 µg/ml LPS added after collecting and replacing the medium every 24 h (at time points t_0, t_{24}, t_{48}) from day 11 to day 14. To deplete microglia, PLX5622 (10 µM) was continuously added to the culture medium starting from the seventh day until the end of the culture period. After the first, second, and third LPS stimulations, the culture medium was collected and analyzed for cytokine levels. (a) TNF-α, (b) IL-1β, and (c) IL-6 in the culture medium after each LPS challenge (n= 2 independent cultures per group; 3 animals/culture). The results are presented as the Mean ± Standard Error of Mean (Mean ± SEM).

Supplementary Figure 3

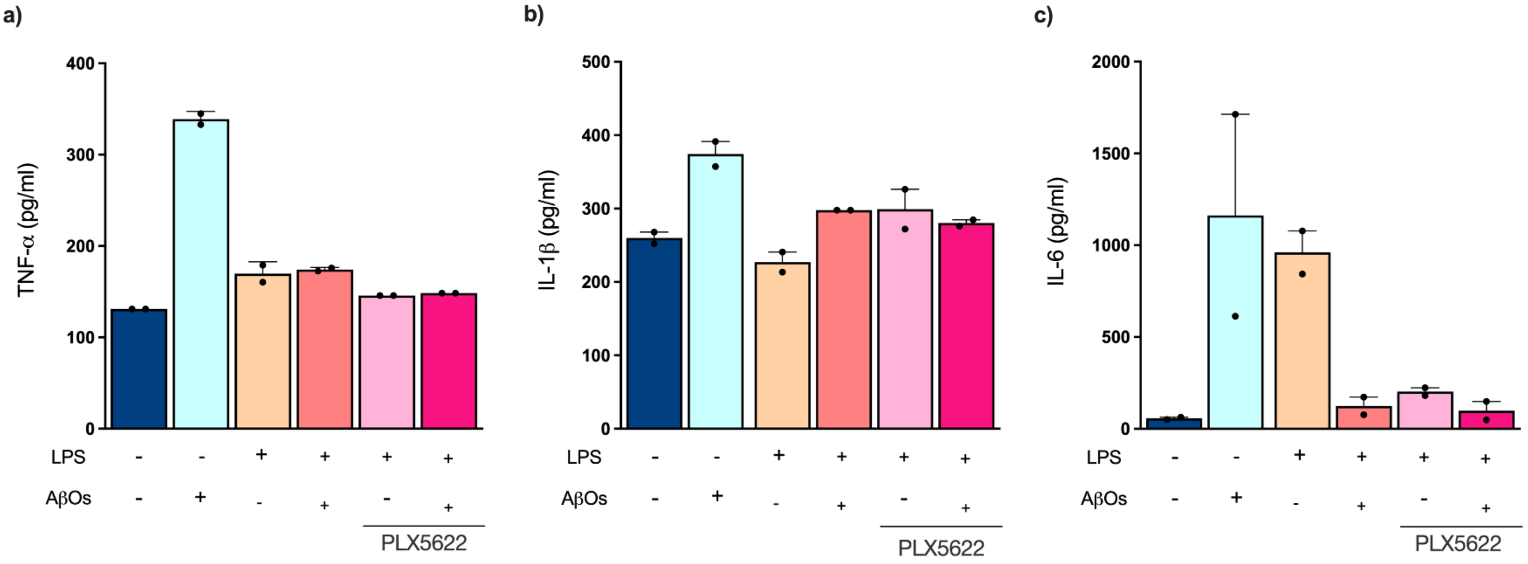

Supplementary Figure 3: Depleting microglia eliminates the elevated levels of inflammatory cytokines induced by LPS or AβOs. PLX5622 (10 µM) was continuously added to the culture medium starting from the seventh day until the end of the culture period. The cultures were repeatedly challenged with LPS, and 72 h after the first LPS stimulation, were exposed to AβOs. The cytokine levels were analyzed in the culture medium 24 h after AβOs exposure. (a) TNF-α, (b) IL-1β, and (c) IL-6 levels in the culture medium 24 hours after exposure of the cultures to AβOs (n= 2 independent cultures per group; 3 animals/culture). The results are presented as the Mean ± Standard Error of Mean (Mean ± SEM).

Supplementary Figure 4

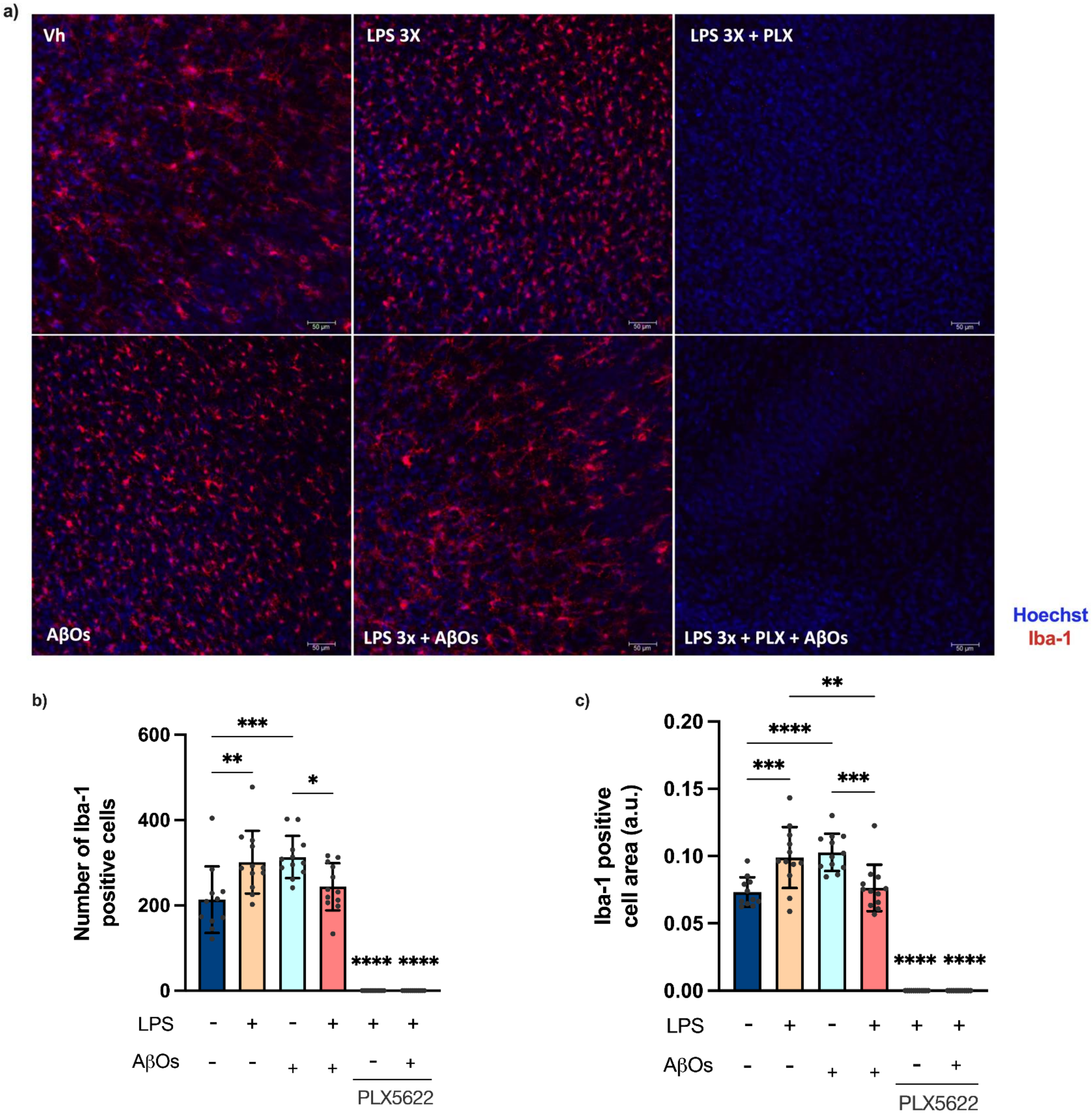

Supplementary Figure 4. Depleting microglia eliminates the increased staining for Iba-1 induced by LPS or AβOs. Microglia were depleted by continuously adding PLX5622 to the culture medium starting from the seventh day until the end of the culture period. After repeated stimulation with LPS, followed by 24 h of AβOs exposure, the staining for Iba1 was performed. (a) Photomicrograph showing microglial cells labeled for Iba-1 (red) and Hoescht (blue). (b) Quantification of the number of Iba-1-positive

cells. (c) Quantification of the area of Iba-1-positive cells. Images were obtained by confocal microscopy. Scale bars: 50 µm. (n= 8/12 images per group). The results are presented as the Mean ± Standard Error of Mean (Mean ± SEM). The data were analyzed using a two-way analysis of variance (ANOVA) followed by the Holm-Sidák test. *p<0.05 **p<0.01 **p<0.001 ****p<0.0001.

Supplementary Figure 5

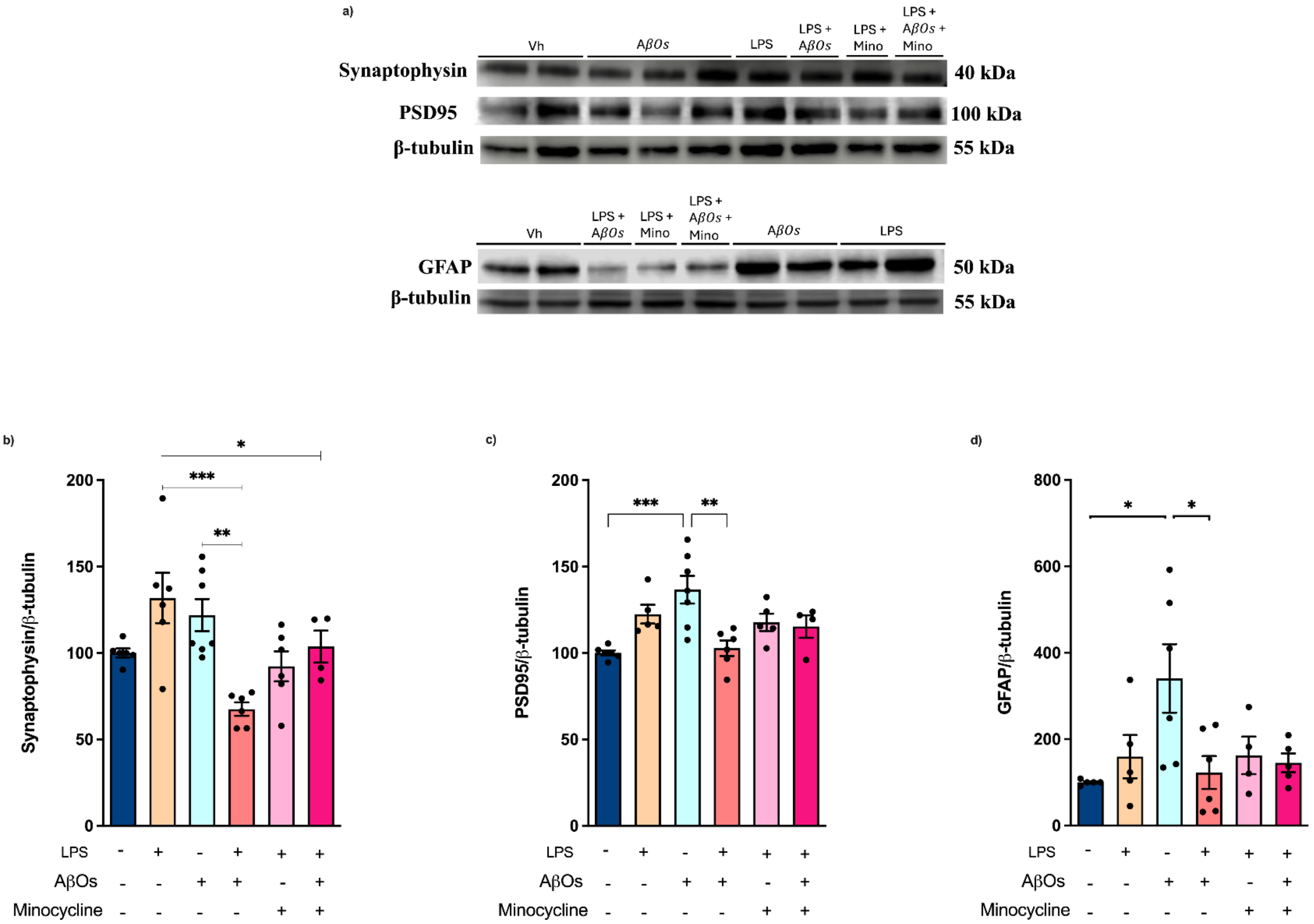

Supplementary Figure 5. Chronic stimulation with LPS prevents changes in the expression of synaptic proteins and GFAP induced by AβOs. Organotypic hippocampal slice cultures were repeatedly stimulated with LPS before being exposed to AβOs. The expression levels of the synaptic proteins synaptophysin and PSD95, along with the Glial Fibrillary Acidic Protein (GFAP), a marker of astrocyte activation, were analyzed by Western blotting 24 h after exposure to AβOs. (a) Representative images of immunoblotting for synaptophysin, PSD95, GFAP, and β-tubulin are shown. Quantification of immunocontent levels for synaptophysin (b), PSD95 (c), and GFAP (d) was normalized by β-tubulin. The y-axis represents the percentage relative to the intensity of the immunoblotting (% of Control). (n= 4-7 animals per group). The results are presented as a percentage of the Mean ± Standard Error of the Mean (Mean ± SEM). The data were analyzed using a two-way analysis of variance (ANOVA) followed by the Holm-Sidák test. *p<0.05; **p<0.01; ***p<0.001.

Figure 5

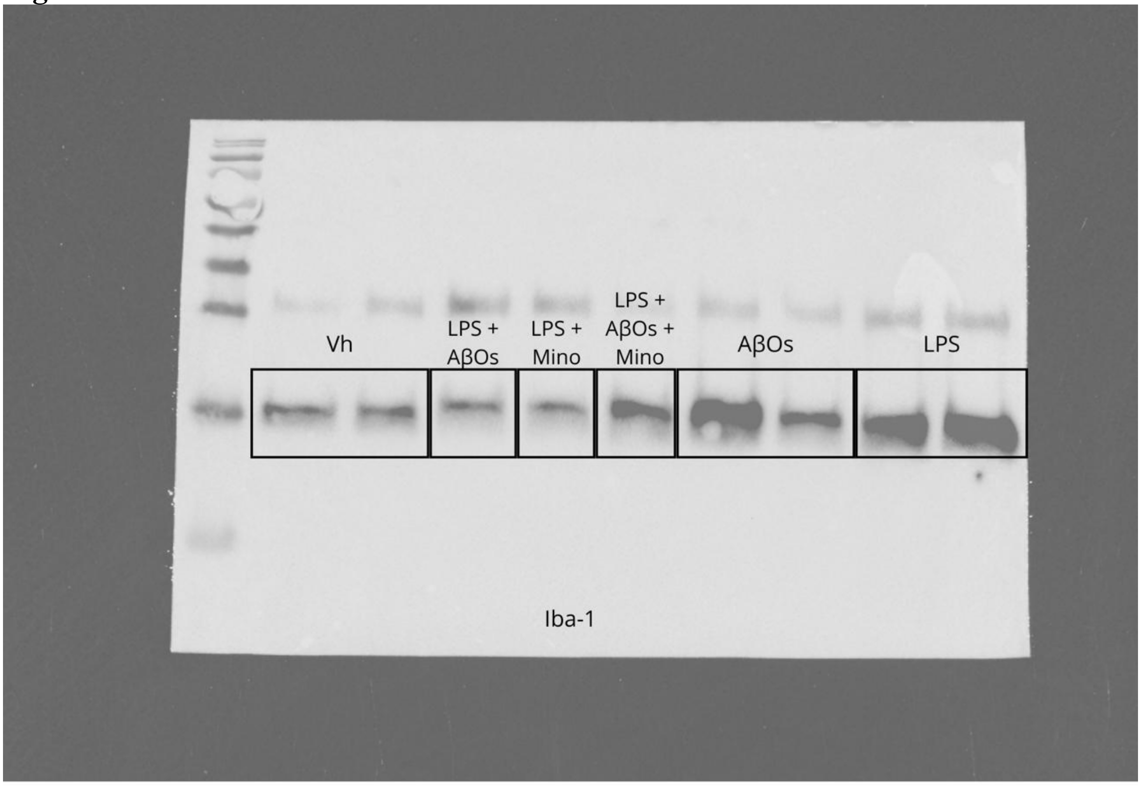
Vh
LPS + AβOs
LPS + Mino
LPS + AβOs + Mino
AβOs
LPS
Iba-1

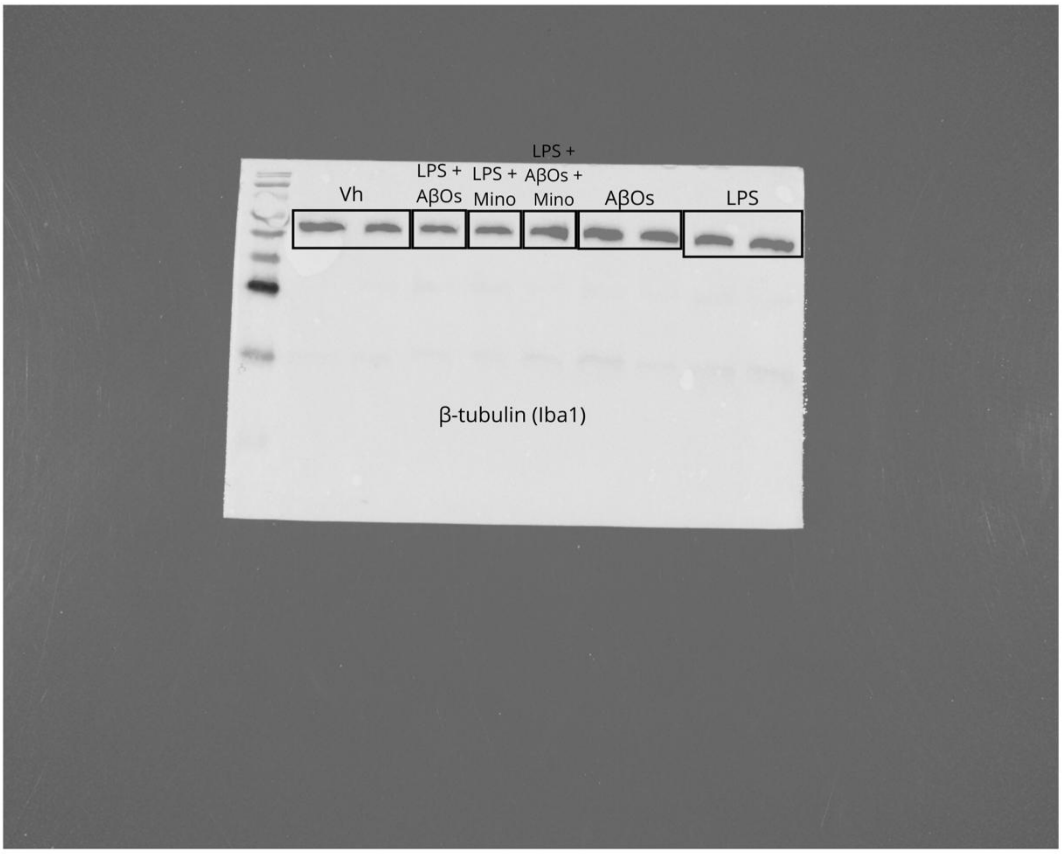
Vh
LPS + AβOs
LPS + Mino
LPS + AβOs + Mino
AβOs
LPS
β-tubulin (Iba1)

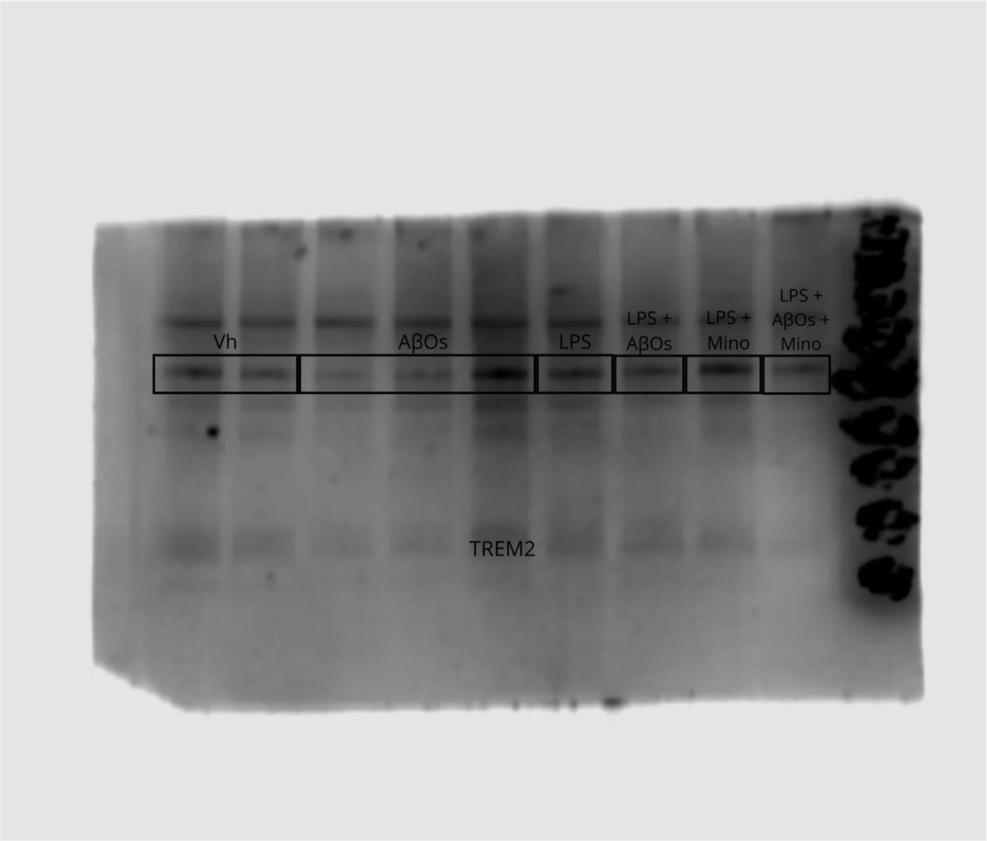
Vh
AβOs
LPS
LPS + AβOs
LPS + Mino
LPS + AβOs + Mino
TREM2

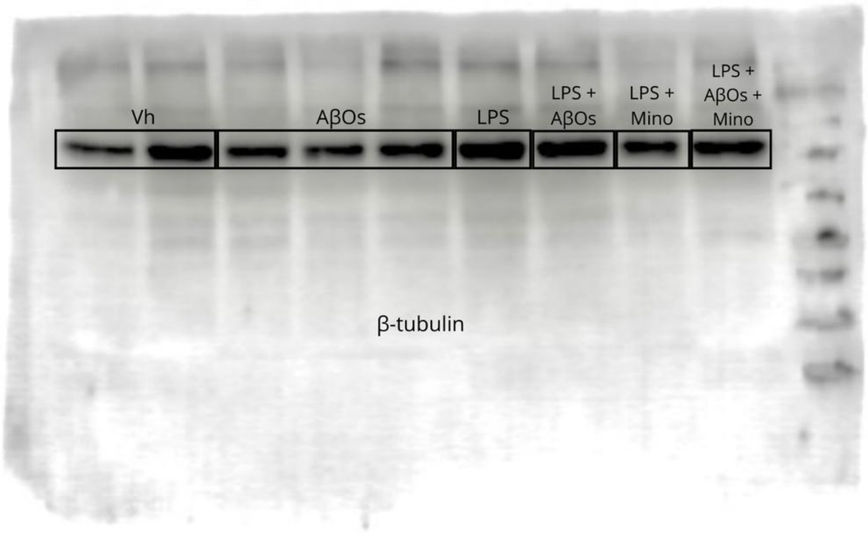
Vh
AβOs
LPS
LPS + AβOs
LPS + Mino
LPS + AβOs + Mino
β-tubulin

Figure 6

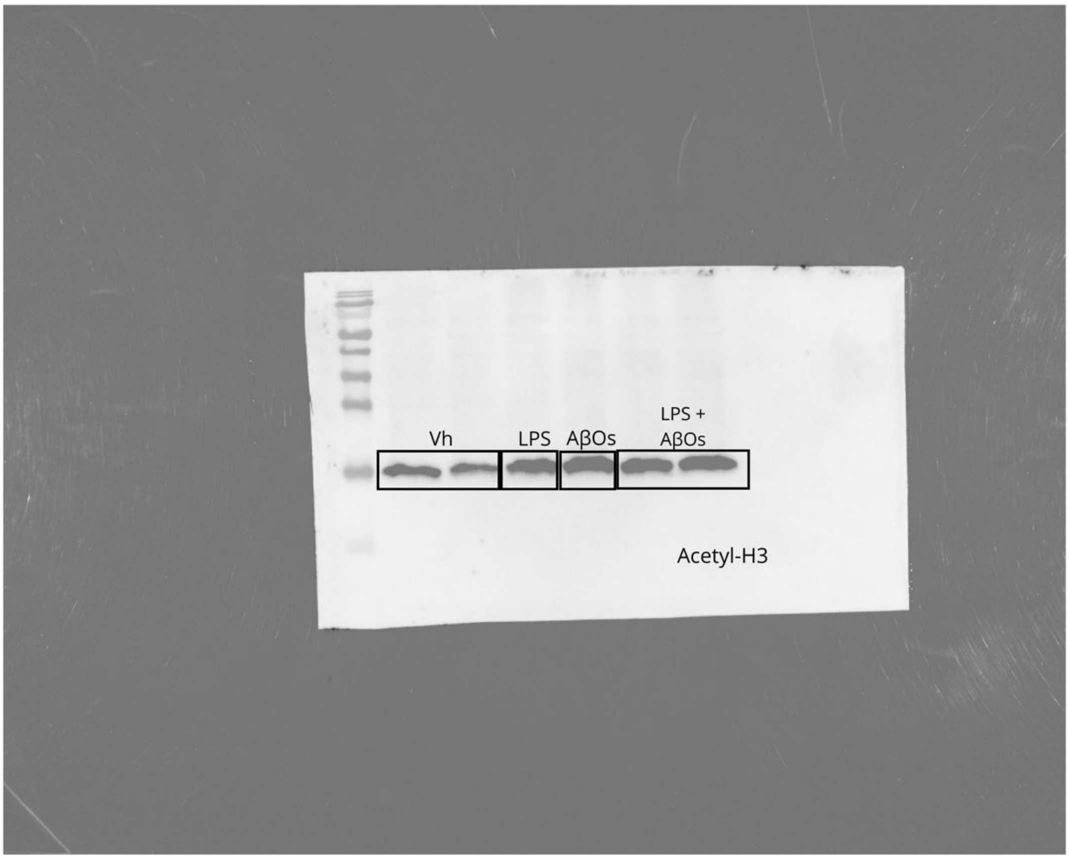

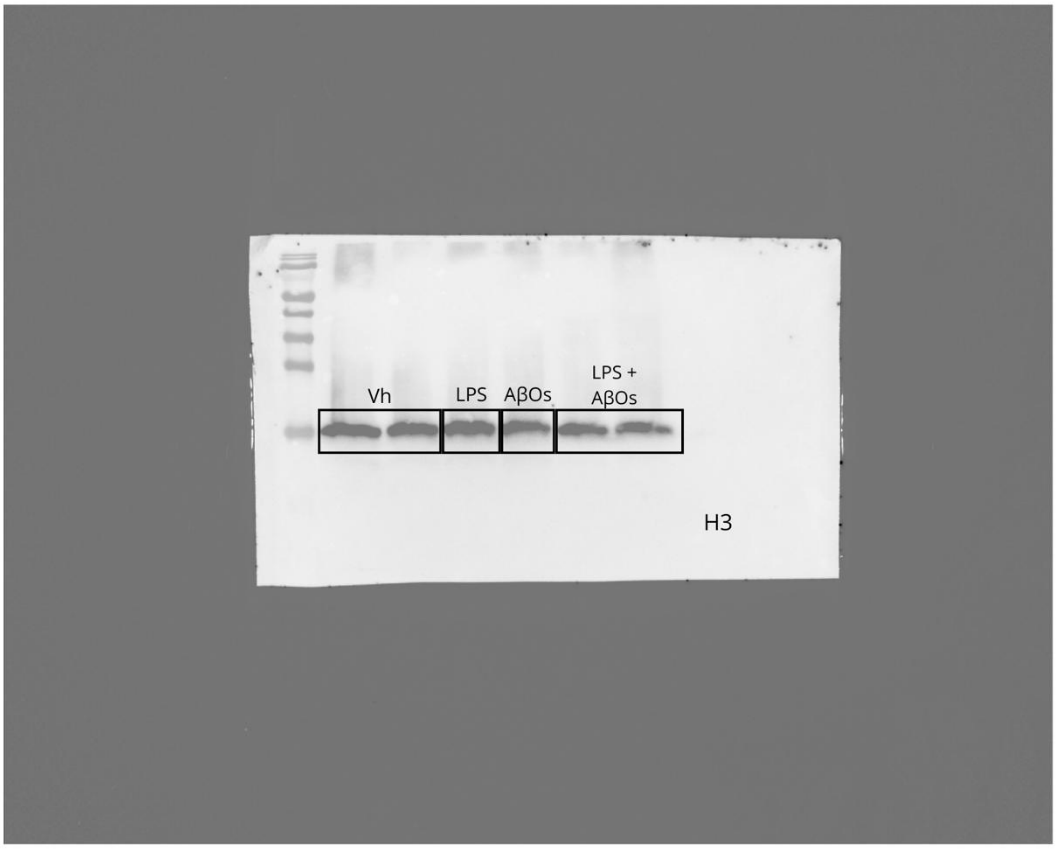

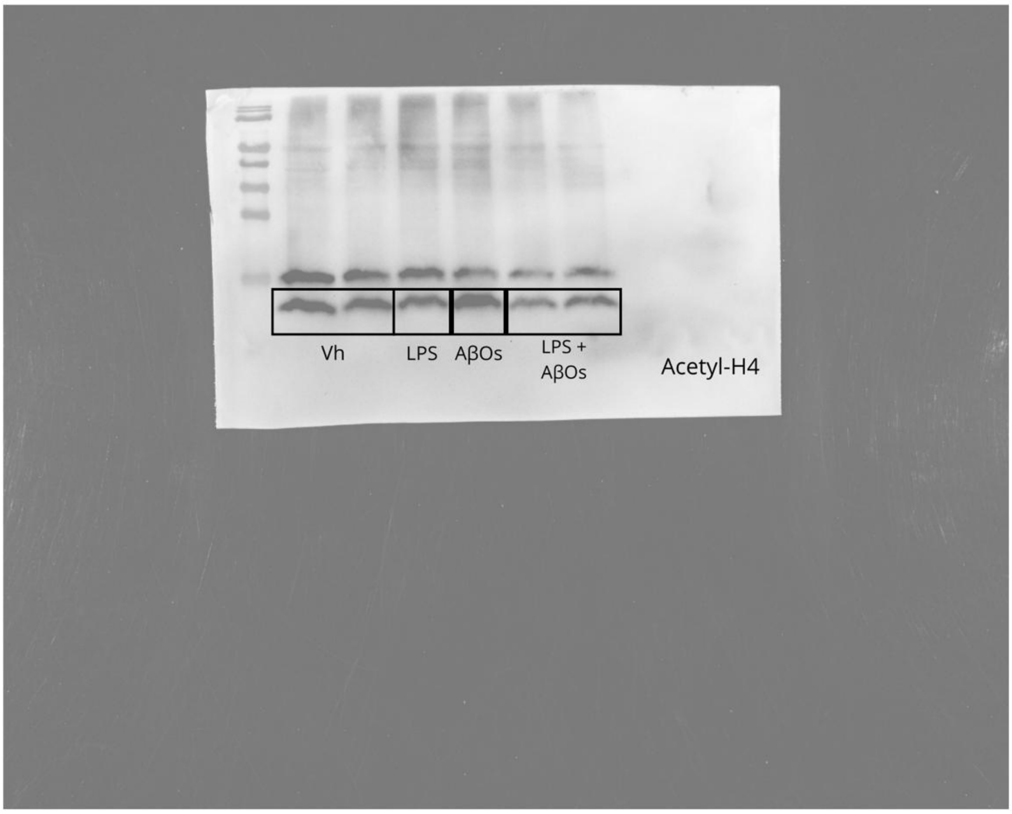
Vh
LPS
AβOs
LPS +
AβOs
Acetyl-H4

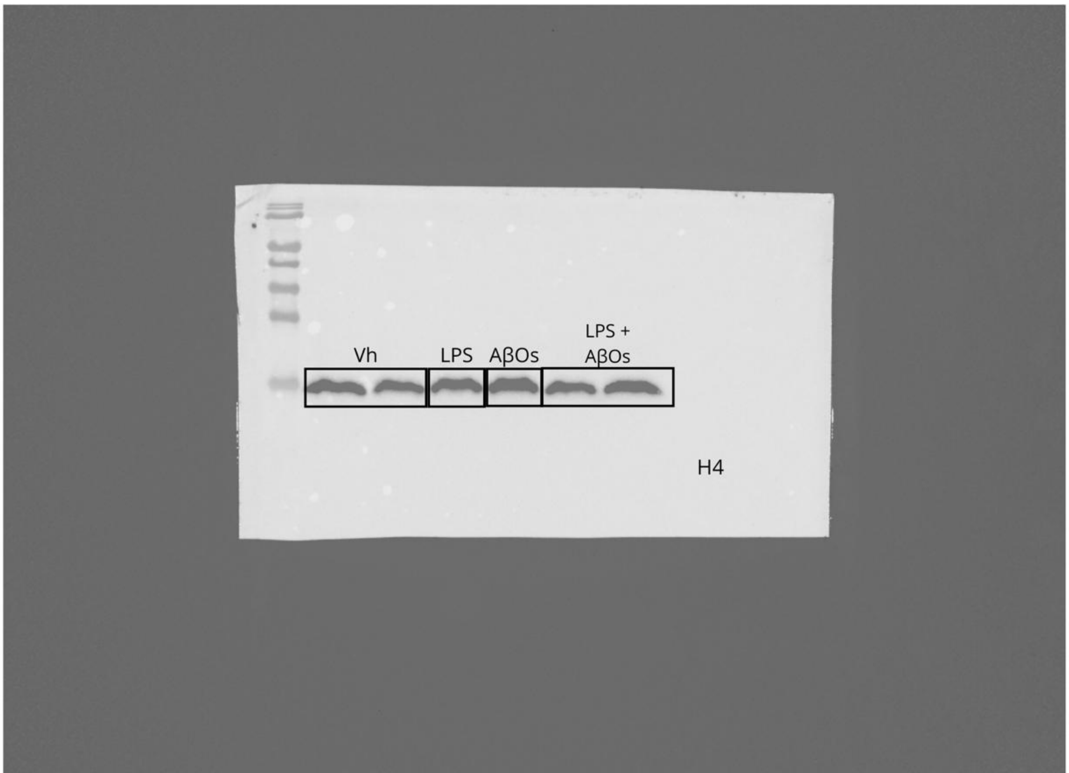
Vh
LPS
AβOs
LPS +
AβOs
H4

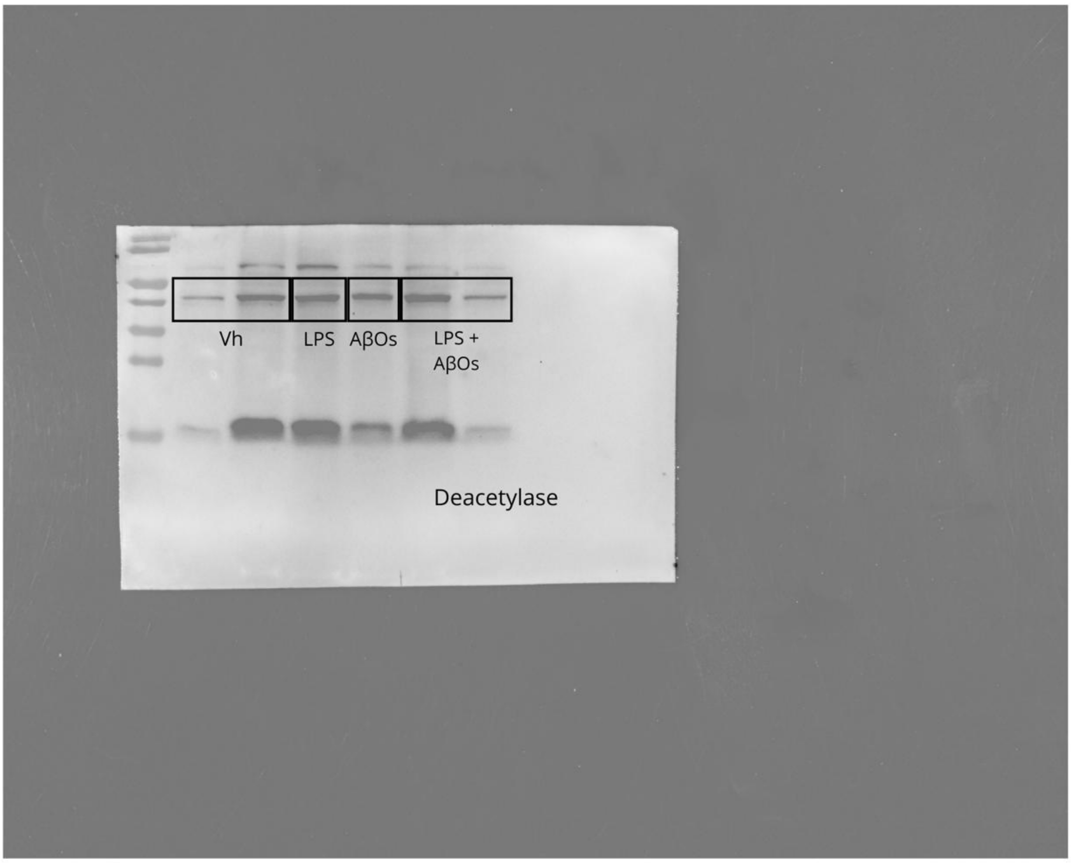
Vh
LPS
AβOs
LPS +
AβOs
Deacetylase

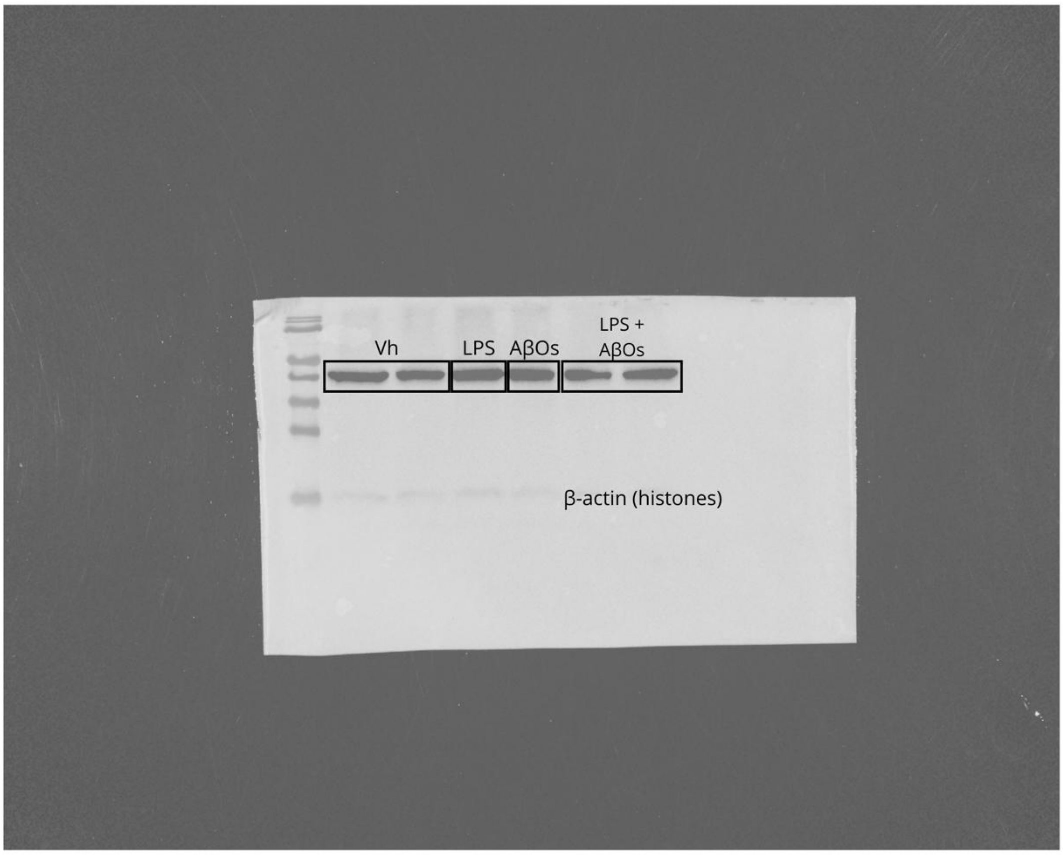
Vh
LPS
AβOs
LPS +
AβOs
β-actin (histones)

Figure 7

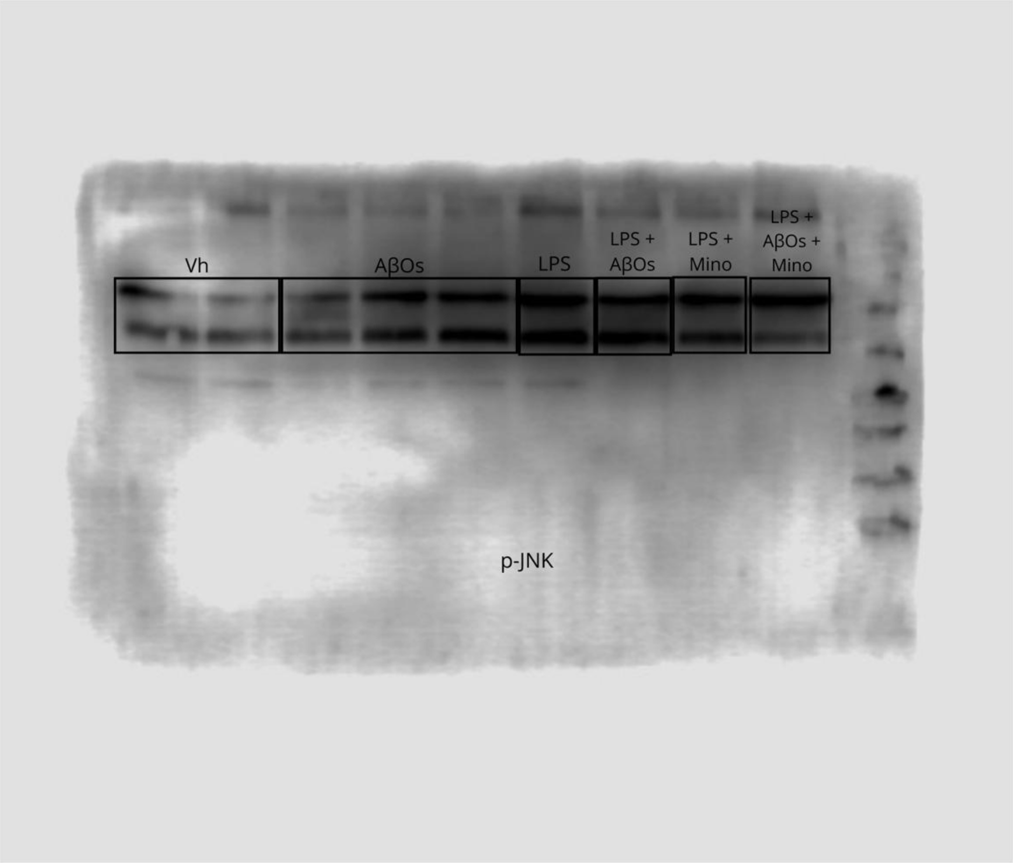

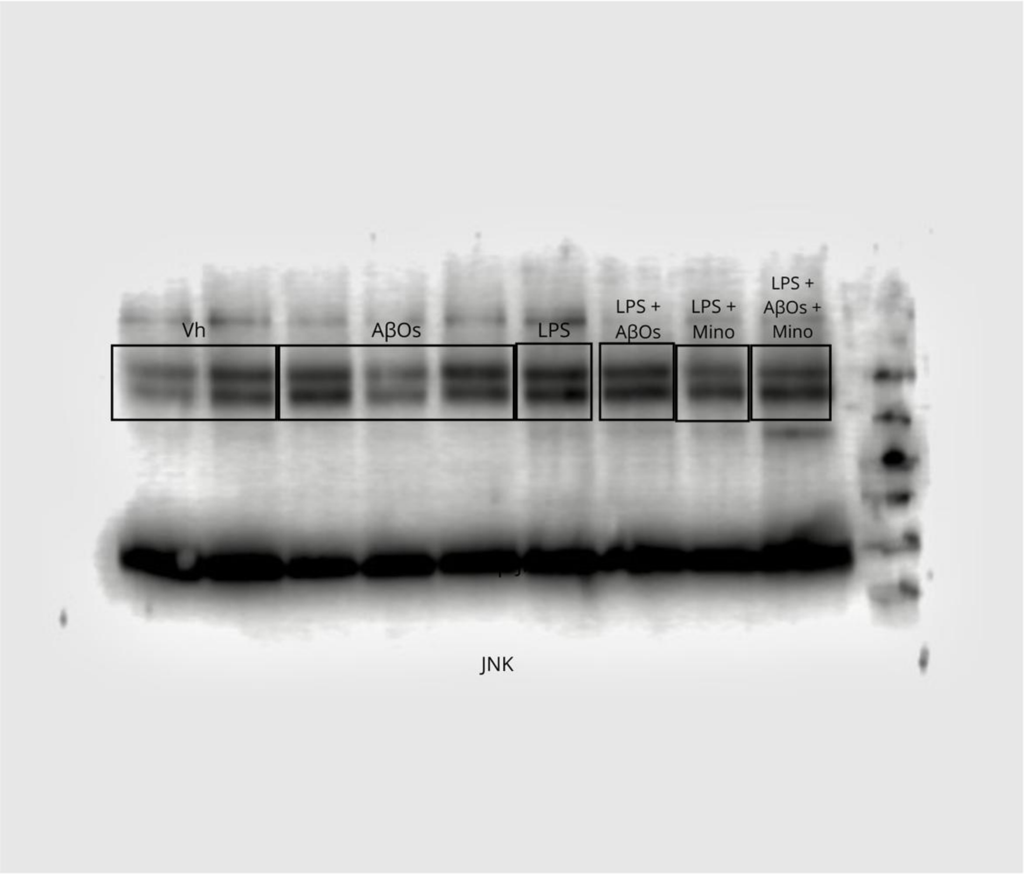
Vh
AβOs
LPS
LPS + AβOs
LPS + Mino
LPS + AβOs + Mino
JNK

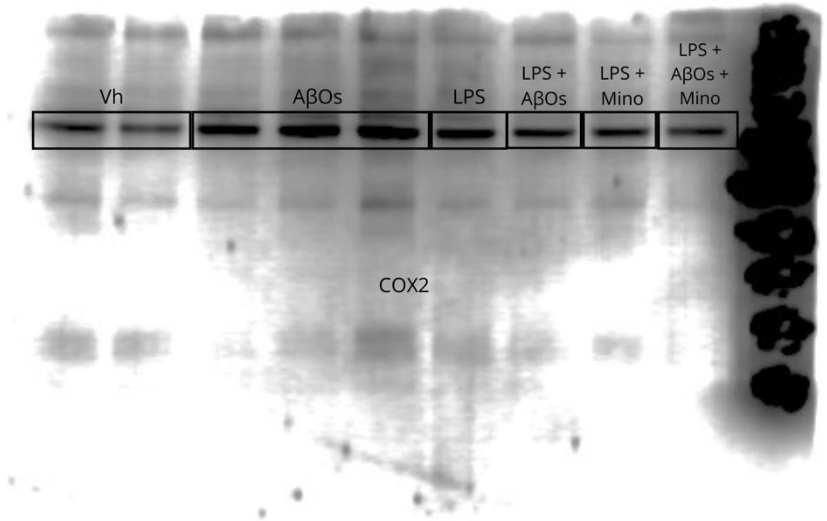
Vh
AβOs
LPS
LPS + AβOs
LPS + Mino
LPS + AβOs + Mino
COX2

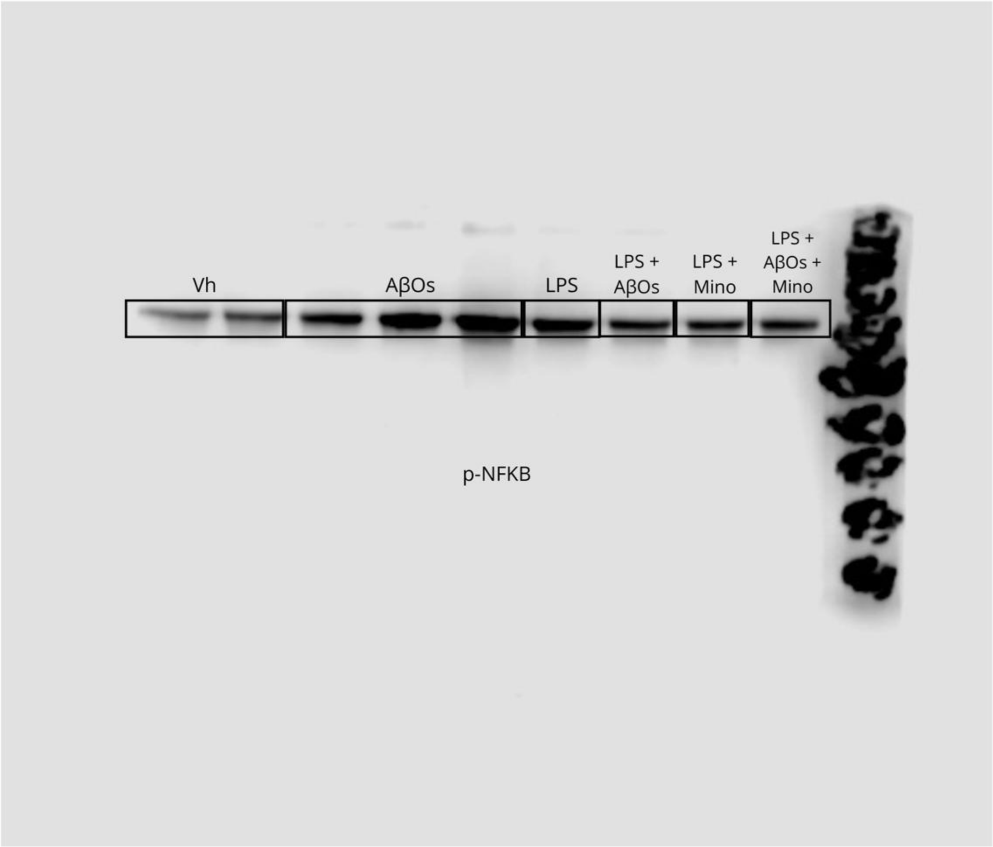
Vh
AβOs
LPS
LPS + AβOs
LPS + Mino
LPS + AβOs + Mino
p-NFKB

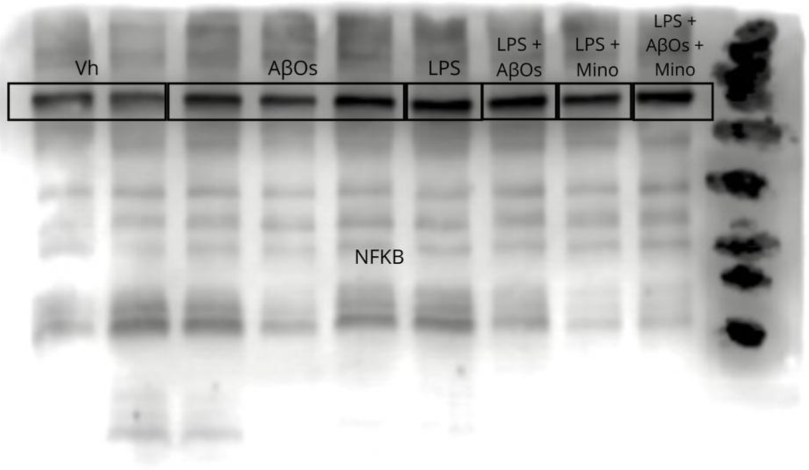
Vh
AβOs
LPS
LPS + AβOs
LPS + Mino
LPS + AβOs + Mino
NFKB

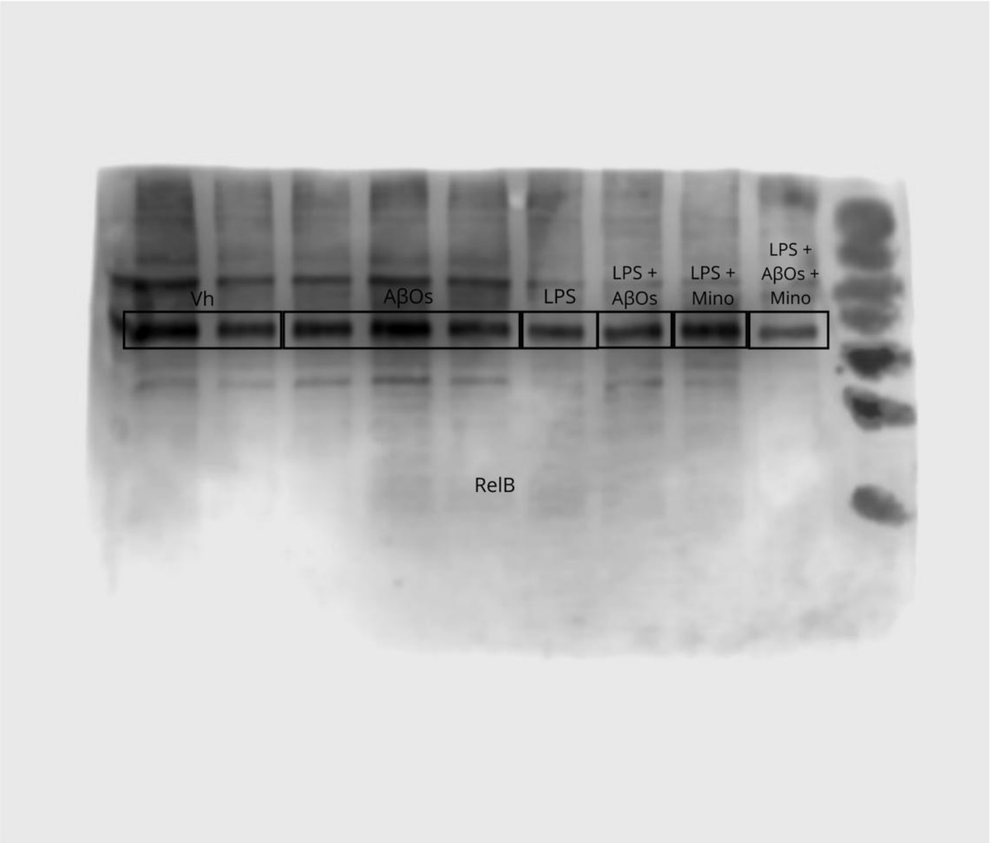
Vh
AβOs
LPS
LPS + AβOs
LPS + Mino
LPS + AβOs + Mino
RelB

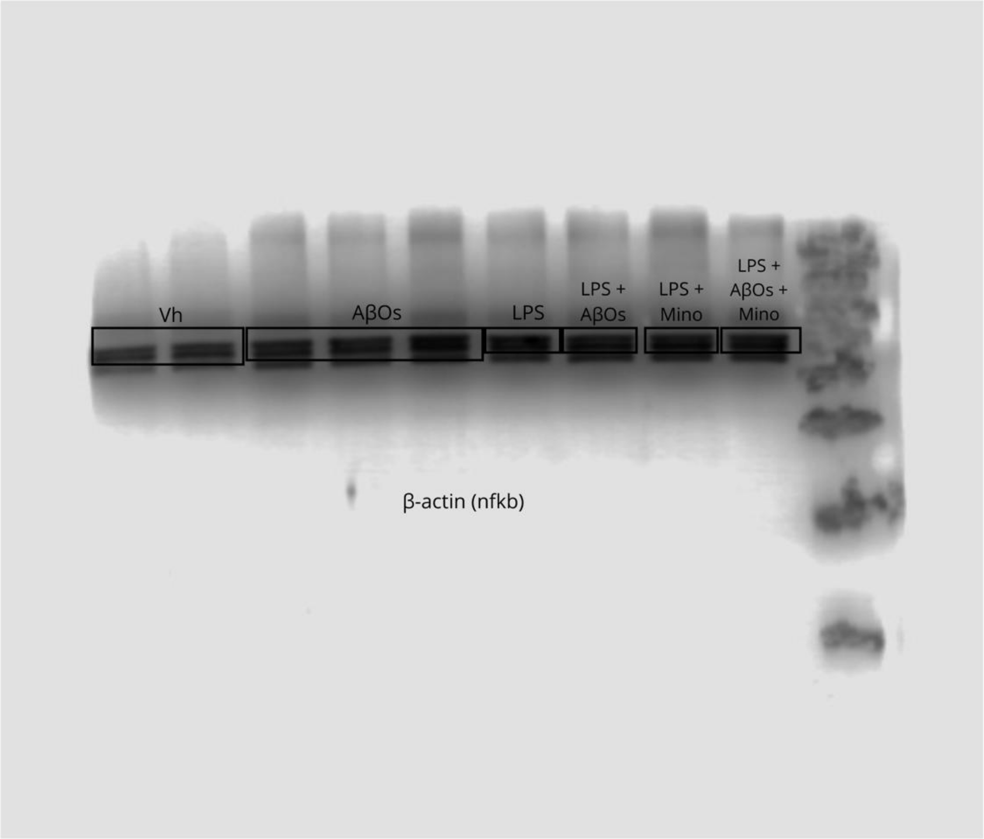
Vh
AβOs
LPS
LPS + AβOs
LPS + Mino
LPS + AβOs + Mino
β-actin (nfkb)

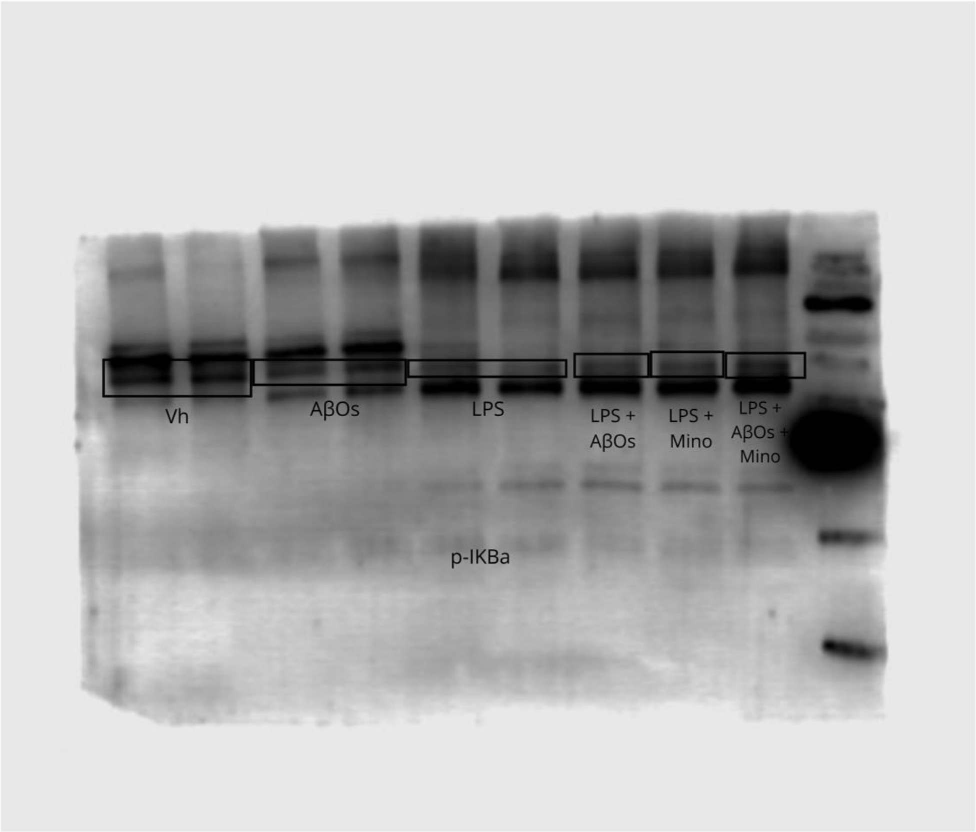
Vh
AβOs
LPS
LPS +
AβOs
LPS +
Mino
LPS +
AβOs +
Mino
p-IKBa

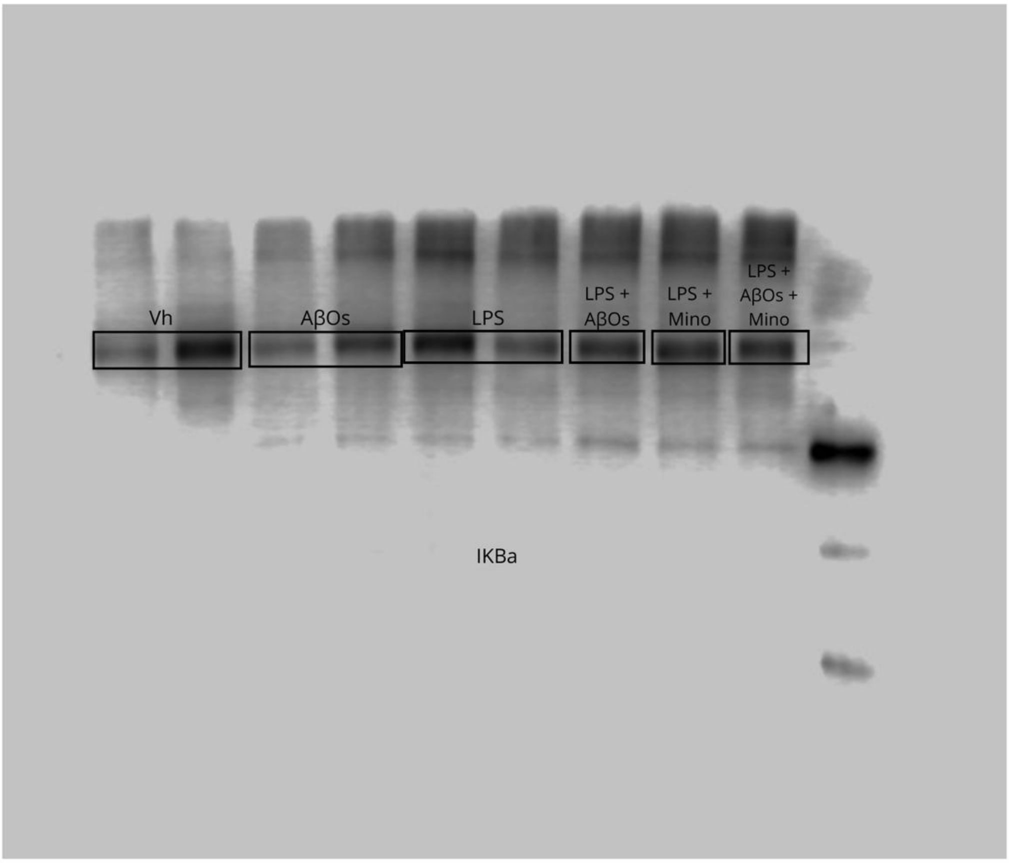
Vh
AβOs
LPS
LPS +
AβOs
LPS +
Mino
LPS +
AβOs +
Mino
IKBa

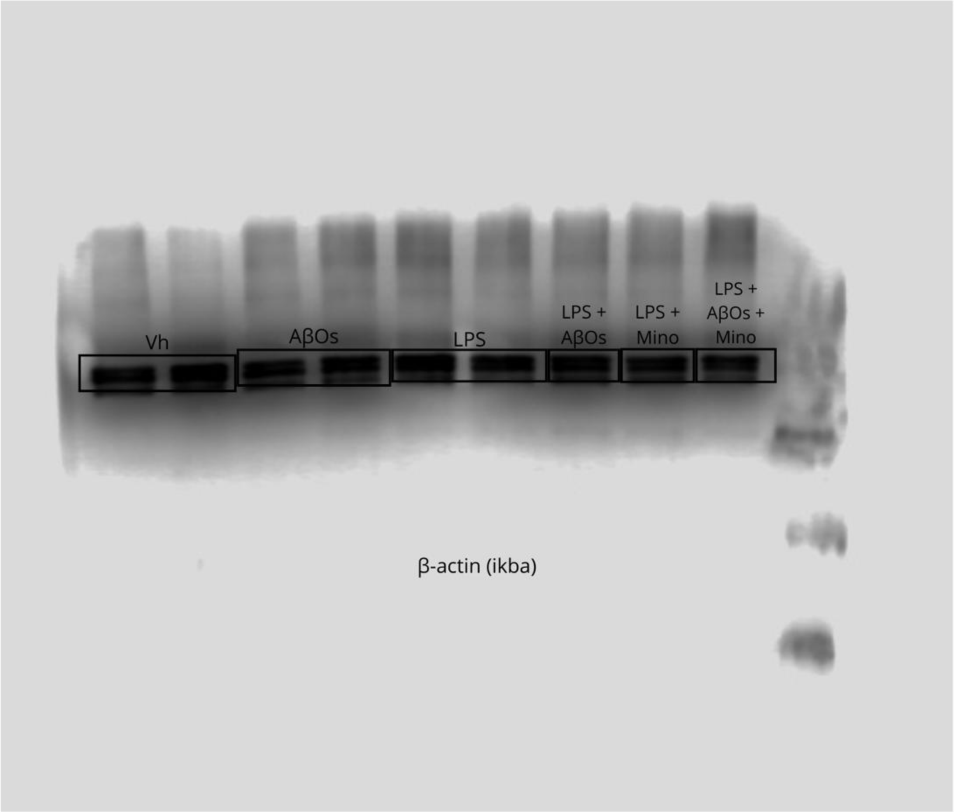
Vh
AβOs
LPS
LPS + AβOs
LPS + Mino
LPS + AβOs + Mino
β-actin (ikba)

Supplementary Figure 5

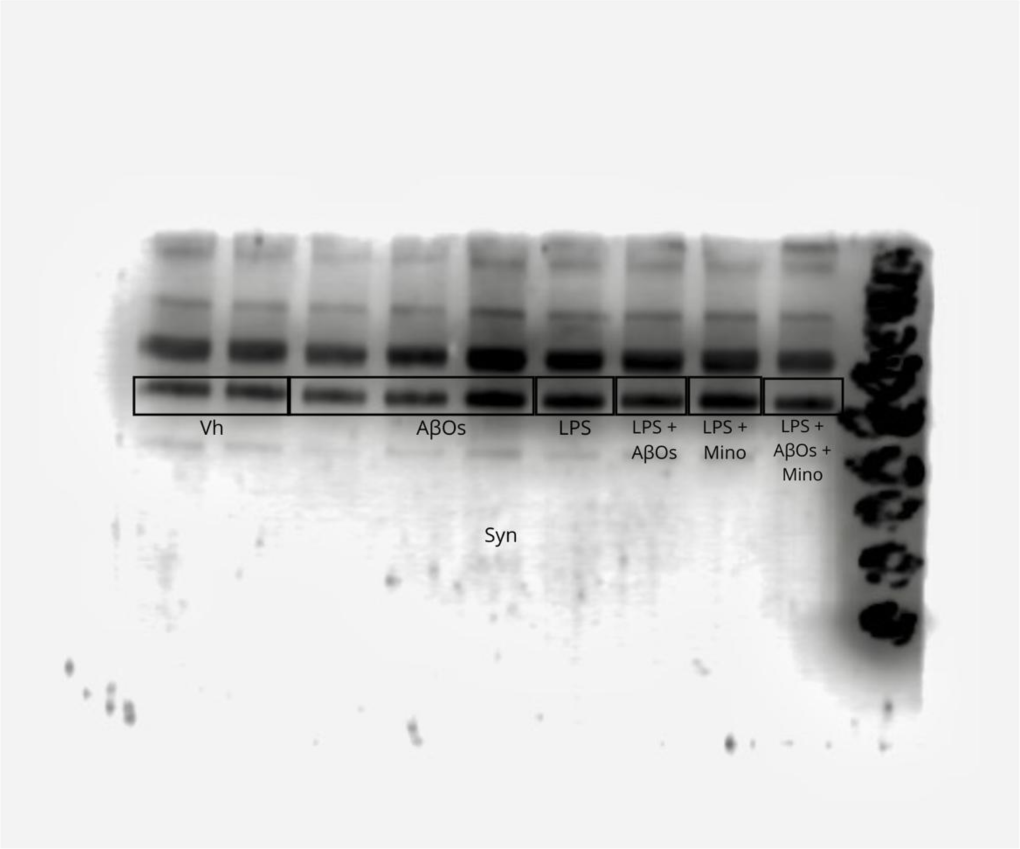

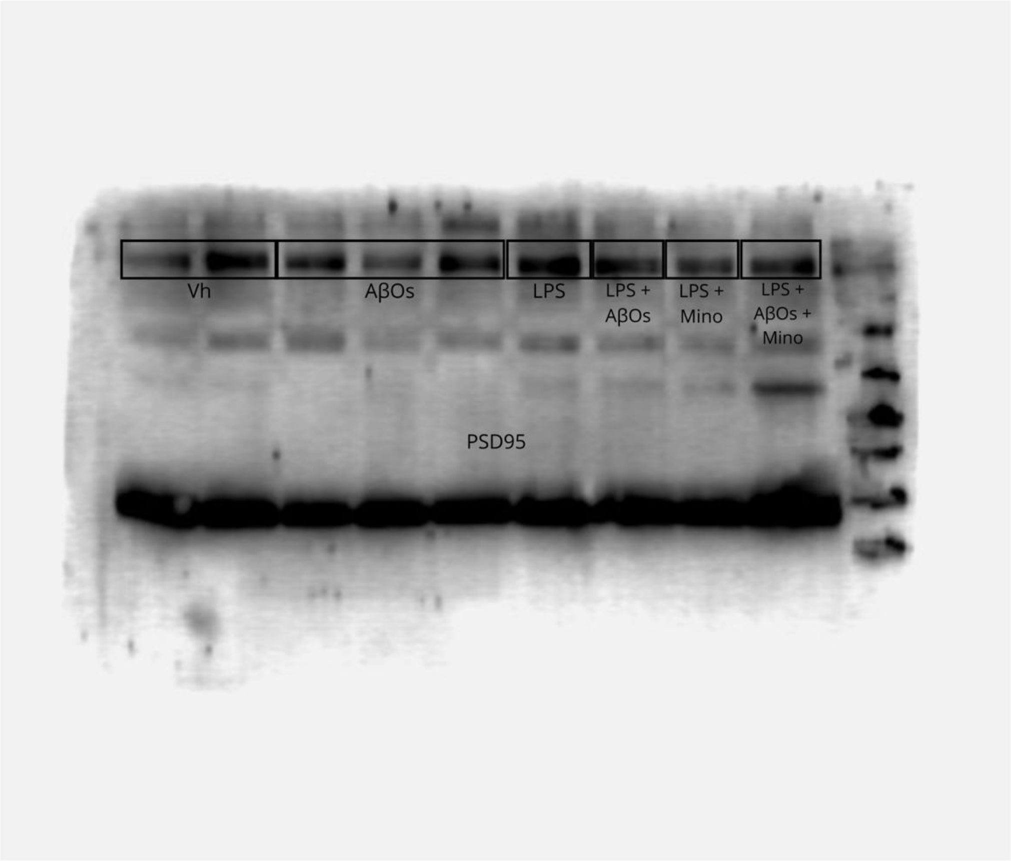

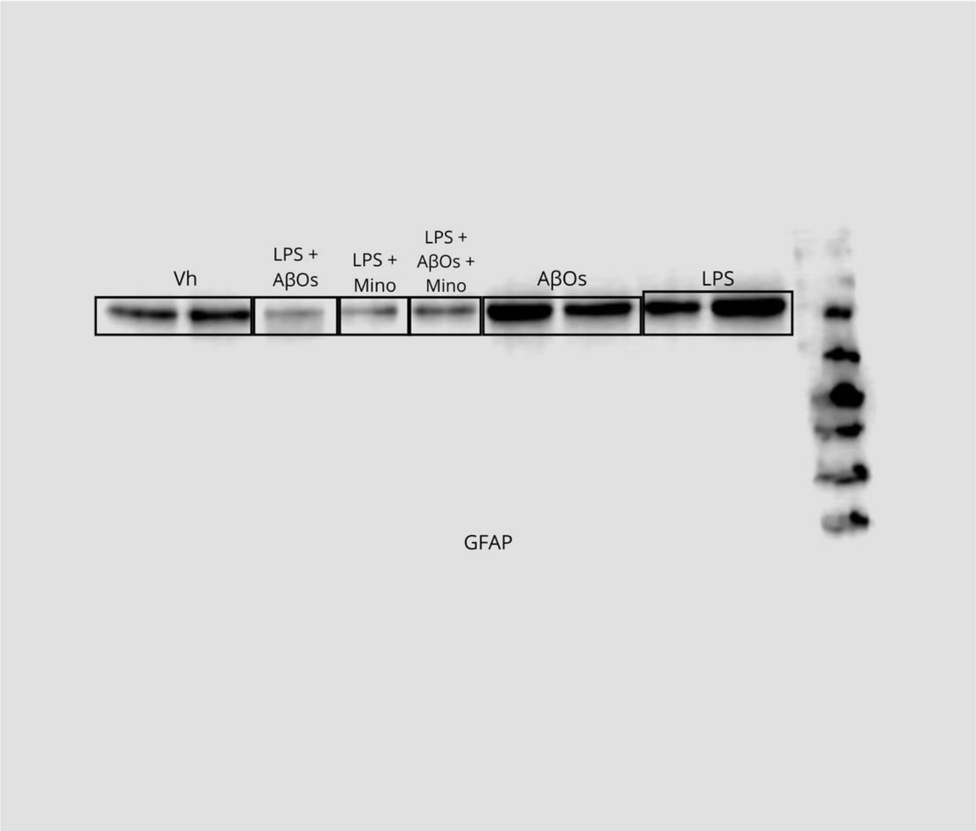
Vh
LPS +
AβOs
LPS +
Mino
LPS +
AβOs +
Mino
AβOs
LPS
GFAP

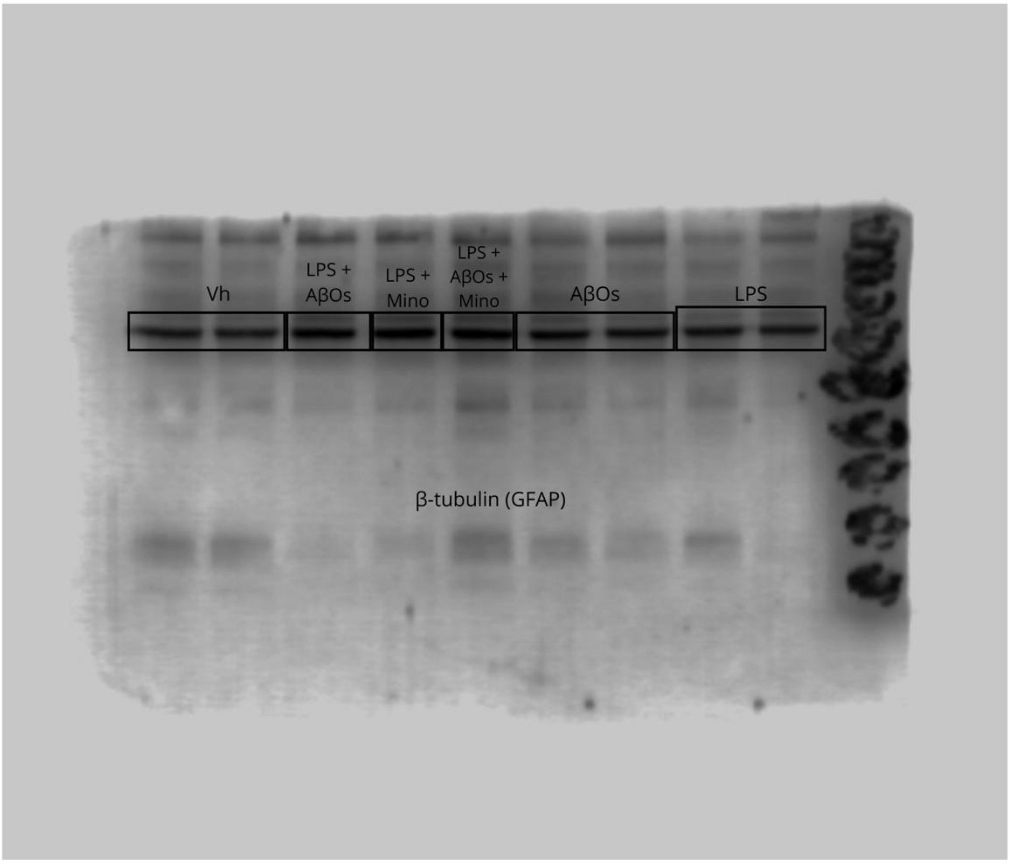
Vh
LPS +
AβOs
LPS +
Mino
LPS +
AβOs +
Mino
AβOs
LPS
β-tubulin (GFAP)
